# *N*-Benzyl Residues as the P1′ Substituents in Phosphorus-Containing Extended Transition State Analog Inhibitors of Metalloaminopeptidases

**DOI:** 10.3390/molecules25184334

**Published:** 2020-09-22

**Authors:** Kamila Janiszewska, Michał Talma, Bartosz Oszywa, Małgorzata Pawełczak, Paweł Kafarski, Artur Mucha

**Affiliations:** 1 Department of Bioorganic Chemistry, Wrocław University of Science and Technology, Wybrzeże Wyspiańskiego 27, 50-370 Wrocław, Poland; kamila.janiszewska.87@gmail.com (K.J.); michal.talma@pwr.edu.pl (M.T.); pawel.kafarski@pwr.edu.pl (P.K.); 2 Institute of Chemistry, University of Opole, Oleska 48, 45-052 Opole, Poland; bartoszoszywa@interia.pl (B.O.); Malgorzata.Pawelczak@uni.opole.pl (M.P.)

**Keywords:** organophosphorus compounds, peptide analogs, enzyme inhibitors, ligand-enzyme interactions, molecular modeling and docking

## Abstract

Peptidyl enzyme inhibitors containing an internal aminomethylphosphinic bond system (P(O)(OH)-CH_2_-NH) can be termed extended transition state analogs by similarity to the corresponding phosphonamidates (P(O)(OH)-NH). Phosphonamidate pseudopeptides are broadly recognized as competitive mechanism-based inhibitors of metalloenzymes, mainly hydrolases. Their practical use is, however, limited by hydrolytic instability, which is particularly restricting for dipeptide analogs. Extension of phosphonamidates by addition of the methylene group produces a P-C-N system fully resistant in water conditions. In the current work, we present a versatile synthetic approach to such modified dipeptides, based on the three-component phospha-Mannich condensation of phosphinic acids, formaldehyde, and *N*-benzylglycines. The last-mentioned component allowed for simple and versatile introduction of functionalized P1′ residues located on the tertiary amino group. The products demonstrated moderate inhibitory activity towards porcine and plant metalloaminopeptidases, while selected derivatives appeared very potent with human alanyl aminopeptidase (*K*_i_ = 102 nM for **6a**). Analysis of ligand-protein complexes obtained by molecular modelling revealed canonical modes of interactions for mono-metallic alanyl aminopeptidases, and distorted modes for di-metallic leucine aminopeptidases (with C-terminal carboxylate, not phosphinate, involved in metal coordination). In general, the method can be dedicated to examine P1′-S1′ complementarity in searching for non-evident structures of specific residues as the key fragments of perspective ligands.

## 1. Introduction

Phosphonamidate peptide analogues, compounds that comprise a direct nitrogen-to-phosphorus bond (-P(O)(OH)-NH-, P[NH]) introduced instead of an amide bond, have been reported as exceptionally active inhibitors of metalloproteases [1]. For example, pseudotripeptide Cbz-PheP[NH]Leu-Ala-OH, a competitive thigh-binding inhibitor of thermolysin, which is ranked among the most potent inhibitors of this type, displayed a *K*_i_ value in the picomolar range [2]. It is commonly believed that such an affinity originates from structural and electronic similarity of phosphonamidates to the gem-diolate intermediate of the amide bond hydrolysis, which is stabilized by favorable interactions in the metalloenzyme active sites [3,4]. These interactions include, first of all, specific hydrogen bonds formed by the phosphoryl oxygen atoms with the enzyme resides and complexation with the metal ion(s) but also important contacts provided by the NH group. Consistent with these analogies, phosphonamidate peptides are classified as transition state inhibitors. Accordingly, they have served as invaluable tools in numerous mechanistic and structural studies on metalloproteases [1,4]. Searching for new inhibitors of the M1 microsomal alanyl aminopeptidase (E.C.3.4.11.2, APN) and the M17 cytosolic leucine aminopeptidase (E.C.3.4.11.1, LAP), we also turned our attention to phosphonamidate dipeptide analogs [5]. Although very promising in terms of ligand-protein complementarity, phosphonamidates demonstrated high susceptibility to hydrolysis and thus, they were disregarded [6]. Inclusion of the methylene linker into the P-N system produced extended pseudodipeptides that avoided unwanted characteristics. Aminomethylphosphinates should have also merged advantageous active site interactions of phosphonamidates with the chemical stability of phosphinates. Indeed, such a system appeared effective in the construction of inhibitors for aminopeptidases [7] and urease [8].

**Figure 1:**
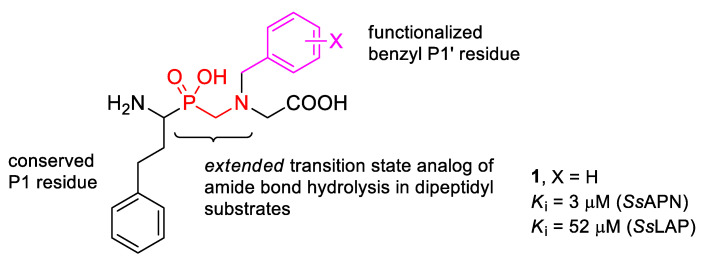
General formula of pseudodipeptides containing an internal aminomethylphosphinate bond system planned for the synthesis. Selected heteroatom-modified/functionalized benzyl P1′ residues are envisaged to be installed as the N-substituents. The activities of the unsubstituted derivative **1** reported previously for porcine enzymes are considered as the reference [7].

In this work, we report the synthesis of pseudodipeptide compounds of the general formula depicted in Figure 1, and preliminary verify their potential to inhibit aminopeptidases. The structure of compounds was based on the conserved 2-phenylethyl P1 side chain, while benzyl P1′ residues were planned to be introduced as N-substituents. The derivative containing unsubstituted benzyl (**1**) was assayed in our previous studies showing an interesting affinity towards porcine APN (*Ss*APN, *Sus scrofa*) and selectivity versus LAP (*Ss*LAP) [7]. Here, we applied heteroatom-modified or functionalized substrates to diversify the P1′ residue. In addition to *Ss*LAP and *Ss*APN, the selected compounds were tested with human alanyl aminopeptidase (*Hs*APN, *Homo sapiens*). A plant aminopeptidase isolated from barley seeds (*Hv*LAP, *Hordeum vulgare* L.) was also included for evaluation of the biological activity.

## 2. Results and Discussion

### 2.1. Chemistry

**Scheme 1:**
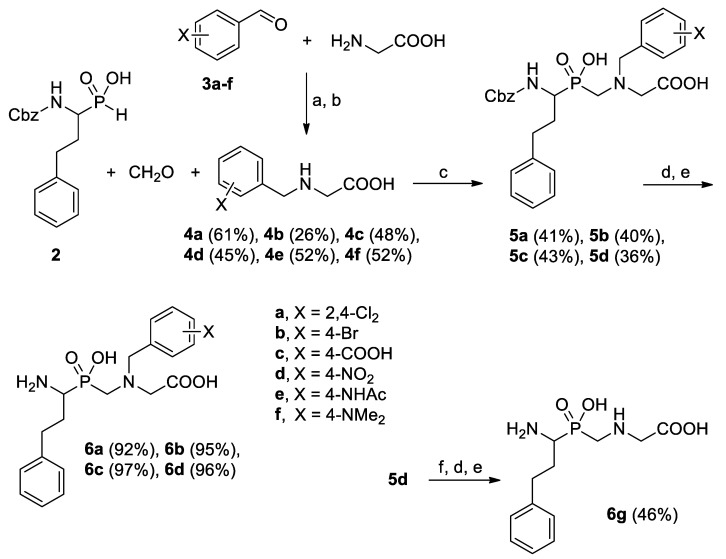
The synthesis of pseudodipeptides containing the aminomethylphosphinic bond system. Reagents and conditions: (a) KOH, NaBH_4_, EtOH, 15 °C, 24 h; (b) pH 4–5; (c) CH_2_O (36–38% aqueous solution, 2.0 eq.), H_2_O/AcOH (1:1, *v*/*v*), HCl_conc_ (1.0 eq.), reflux, 5 h; (d) HBr (33% solution in AcOH), room temperature, 2 h; (e) propylene oxide; (f) SnCl_2_, EtOH, reflux, 3 h.

The three-component Mannich-type condensation of α-aminoalkyl-*H*-phosphinic acid, formaldehyde, and *N*-benzylglycines was used to obtain the target P-C-N system (Scheme 1). The preparation follows the general methods of aminometylation of P-H compounds, such as phosphorous [9] or hypophosphorous acid [10]. The method has been broadly used to obtain multifunctional aminocarboxylic/phospho(i)nic acids as metal complexating and antiscale agents, water softeners, or herbicides [11,12]. Typically, secondary amines or N-substituted amino acids give the products in a higher yield than primary counterparts. Thus, 1-*N*-Cbz-amino-3-phenylpropyl-*H*-phosphinic acid (**2**) [5,13] was heated with an excess of formaldehyde and *N*-benzylglycines **4a**–**f** under acidic conditions (HCl addition). To achieve solubility of the phosphinic acid, the reactions were carried out in a water/acetic acid mixture. The amino acid components **4a**–**f** were synthesized by reductive amination of benzaldehydes **3a**–**f** with glycine and NaBH_4_ [14]. A few different benzaldehydes, namely halogen-containing (Cl, Br) or functionalized (COOH, NO_2_, NHAc, NMe_2_) were selected as the precursors. According to the architecture of the aminopeptidase S′ pockets, which are hydrophobic clefts with functionalities located at the bottom [15], modifications in *para* position were mostly considered. The yield of *N*-benzylation was moderate, but separation straightforward—pure N-substituted glycines crystallized upon adjusting the appropriate pH.

Conveniently, N-protected pseudodipeptide products **5** of the condensation also crystallized clean in the reaction mixture after cooling. However, for substrates **4e** and **4f**, the expected compounds **5e** and **5f** were not separated. The analysis demonstrated debenzylation of the products. The presence of nitrogen-based activating substituents (NHAc and NMe_2_, respectively) increased benzyl susceptibility to acidic hydrolysis in the reaction conditions. Thus, instead of **5e** and **5f**, the unsubstituted dipeptide was obtained. A similar situation of *N*-benzyl cleavage occurred in an attempt of the nitro group reduction in the Cbz pseudodipeptide **5d**. The SnCl_2_-mediated reaction apparently produced a free amino group, which simultaneously sensibilized *N*-benzyl to acidic pH (Scheme 1).

**Figure 2:**
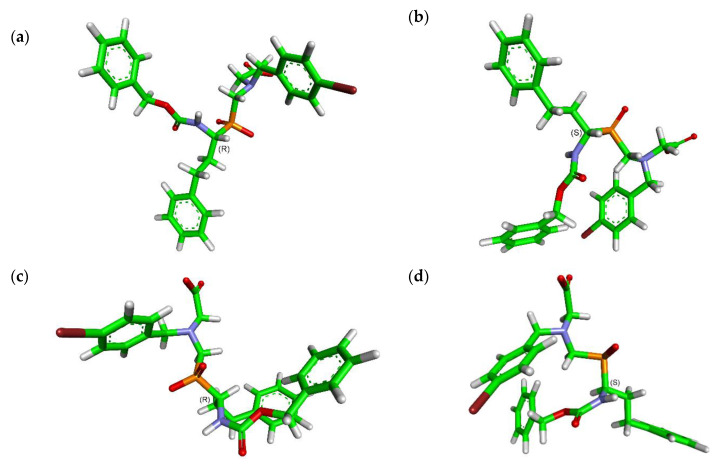
Modeled conformations of *N*-benzyloxycarbonyl-protected 4-Br derivative **4b**, with energies minimized for each stereoisomer. (**a**) *trans*-carbamate/(*R*), ΔG = −266.9 kJ/mol; (**b**) *trans*-carbamate/(*S*), ΔG = −280.8 kJ/mol; (**c**) *cis*-carbamate/(*R*), ΔG = −258.1 kJ/mol; (**d**) *cis*-carbamate/(*S*), ΔG = −269.2 kJ/mol.

Those synthesized and separated N-Cbz products (**5a**–**d**) revealed two sharp resonances in ^31^P NMR spectra and certain doubled signals in ^1^H and ^13^C NMR. This is obviously consistent with *cis*/*trans* isomerism of the carbamate bond. Typically, the *cis* stereoisomer is greatly underrepresented (<10% of relative intensity) and gives a broad ^31^P NMR that can coalesce with the *trans* signal by mild warming [16]. In the case of our compounds, the adopted conformations seemed to be more stabilized. To have an insight into the details, we studied these conformations by molecular modelling and the results for the 4-Br derivative **5b** are depicted in Figure 2.

Energy minimization was performed for each of the four stereoisomers, namely combinations of *cis*/*trans* carbamate and (*R*/*S*) α carbon atom configurations. As expected, the *trans* forms reveals somewhat lower energies than the *cis* ones; however, this is only within the pairs of the same absolute configuration. Interestingly, both (*R*) enantiomers showed a tendency to adopt “open” arrangements without any significant intramolecular interactions that reflected with higher energies. In the contrary, the (*S*) enantiomers were assumed to fold partially and thus to be energetically privileged. π-π interactions between the 4-bromophenyl ring and the carbamate, characteristically located in the structure of studied compounds, were proposed as being responsible for folding.

N-Cbz removal gave deprotected peptides **6a**–**d** and **6g** in the form of hydrobromides that were additionally treated with propylene oxide to remove HBr. These final steps proceeded practically quantitatively. The whole procedure, although not high yielding (not optimized), gave target materials in a simple and non-problematic manner. Each step (reductive amination, condensation, and deprotection) produced solid compounds for which purification was not demanded. The final pseudodipeptide products were fully characterized (NMR and MS spectra are given in the Supplementary Materials) and subjected to enzymatic assays.

### 2.2. Enzyme Inhibition

The synthesized extended transition state analogs were tested with porcine, human, and plant M1 and M17 aminopeptidases. Mammalian leucine (M1, *Ss*LAP) and alanyl aminopeptidases (M1, *Ss*APN and *Hs*APN) were targeted as inhibition of these enzymes with phosphorus-modified dipeptide analogues was probably the most thoroughly studied [17,18]. LAP and APN are multifunctional broad-band specificity aminopeptidases of known structures, mechanisms of action, and recognized therapeutic potential [19,20,21]. To discuss the selectivity issues, leucine aminopeptidase isolated from barley seeds (*Hv*LAP) [22] was also included in these studies.

The synthesized compounds were found moderate to good competitive inhibitors (Table 1). The inhibition constants strongly varied depending on the enzyme and the organism they originated from. The lowest values of *K*_i_ were found for mono-zinc alanyl aminopeptidases. For the porcine ortholog, it ranged between 5.8 and 34 µM. A halogen-substituted benzyl fragment of **6a** and **6b** gave rise to the most effective inhibition. Nevertheless, the results did not outscore the potency evidenced for the reference compound (**1**) bearing N-unsubstituted benzyl as the P1′ side chain. Introduction of either 4-COOH or 4-NO_2_ caused a certain decrease in the activity of **6c** and **6d**. Any N-substituent appeared profitable as compound **6g** lacking such a residue displayed several-fold diminished inhibitory activity.

Selected derivatives were additionally tested with human alanyl aminopeptidase (*Hs*APN). Compounds **6a**–**d** appeared much more potent than for *Ss*APN although the overall structure–activity relationship pattern remained conserved. Halogen-modified **6a** and **6b** gave the lowest inhibition constants (*K*_i_ = 0.10 and 0.13 µM, respectively). 4-NO_2_ analog (**6d**) was only two-fold, while 4-COOH compound (**6c**) was approximately one order of magnitude less potent, yet with *K*_i_ still at a low micromolar level.

Di-metallic leucine aminopeptidases were inhibited by the developed compounds to a lower extent than APNs. For porcine kidney LAP, only 4-carboxy derivative **6c** appeared to be twice more potent than **1** and equipotent to the N-debenzylated compound (**6g**). It can be stated that for this, enzyme installation of the P1′ fragment on the internal amino group is not profitable. Contrarily, *N*-benzylation was favorable for barley seeds LAP. All the substituted compounds (**6a**–**d**) were more potent (by 2–13-fold) compared to non-modified **6g**. **6d** (modified with 4-NO_2_) was the best within this series, with *K*_i_ = 13 µM.

Discussing the selectivity issues of the structure-activity relationship, installation of halogen-based benzyl substituents provided much more potent inhibitors of APN versus LAPs. In particular, **6b** showed a significant selectivity ratio. The compound was among the best inhibitors found for APNs, while the *K*_i_ values for both LAPs were one to two orders of magnitude higher. The carboxylate-modified derivative appeared a moderate but universal ligand, with comparable constants for three of four aminopeptidases. The electron-withdrawing 4-NO_2_ group worked well in the cases of APNs and *Hw*LAP.

### 2.3. Molecular Modeling

Interactions of the synthesized inhibitors with the active centers of the aminopeptidases were further studied by molecular modeling and docking. The binding mode of the most potent compounds was found specific for each particular aminopeptidase. For porcine and human APNs, a large and hydrophobic catalytic site allowed canonical interactions, which means coordination of the catalytic zinc ion with phosphinate and inclusion of the P1 (phenylethyl) and P1′ residues in the corresponding enzyme pockets. Contrarily, this typical binding pattern was distorted for both LAPs. In these cases, the C-terminal carboxylate took the role of the metal chelator. Although the compounds were assayed as the racemic mixtures, the stereochemical aspects were taken into consideration in modeling. In general, it was evidenced that both enantiomeric forms are capable of binding to the enzymes, apparently because of the conformational flexibility of the ligand molecules. This case is presented in detail for **6b** and *Ss*APN. Nevertheless, the interaction network is usually privileged (in term of the free binding energy) for the (*R*) enantiomer (consistent with l relative configuration); thus, (*R*) ligand complexes are exclusively discussed for other aminopeptidases.

**Figure 3:**
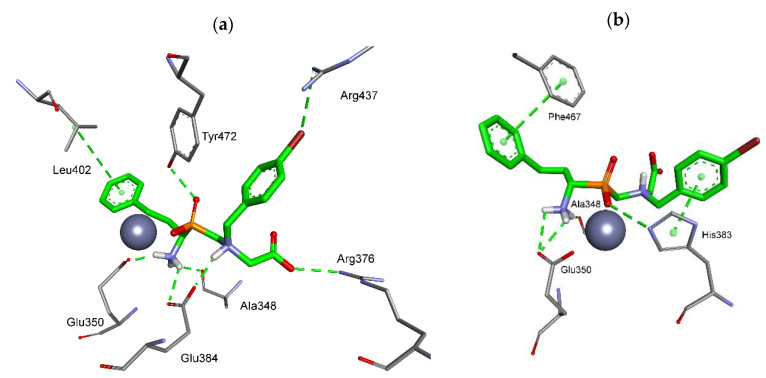
Modeled binding mode of the (*R*) (**a**) and (*S*) enantiomer (**b**) of phosphinic dipeptide analog **6b** containing a 4-bromobenzyl P1′ fragment in the active center of porcine aminopeptidase N (PDB: 4FKE) [23]. Amino acid residues of the inhibitor and enzyme are shown as sticks, while the zinc ion is shown as a gray sphere. Hydrogen bonds and nonpolar interactions are shown as thin dashed lines.

The best poses found for the enantiomers of pseudodipeptide **6b** showed typical monodentate coordination of the zinc ion with phosphinate and convenient inclusion of the side chains in the *Ss*APN binding pockets (Figure 3). The (*R*) enantiomer showed a set of interactions that more specifically resembled the transition state of cleavage of natural substrates. Accordingly, one of the phosphinate oxygen atoms forms the hydrogen bond with the hydroxyl group of Tyr472 (2.52 Å), the residue that is considered to be involved in stabilization of the gem-diolate in native hydrolysis (Figure 3a). The position of the N-terminal amino group is fixed by several contacts with neighboring residues, in particular with carboxylates of Glu350 (1.97 Å) and Glu384 (2.38 Å). In addition, C-terminal functionality of ligand (*R*)-**6b** forms a salt bridge with the guanidino group of Arg376 (2.96 Å). Protonation of the internal tertiary amino group provides an opportunity of hydrogen bonding with one of the oxygen atoms of Glu384 carboxylate (2.01 Å). The most significant hydrophobic contacts involve stacking of P1 phenylethyl with Leu402. Bromine atom is presumably engaged in a weak hydrogen bond with the guanidine group of Arg437 (3.39 Å) (Figure 3a).

For the opposite enantiomer, the arrangement of polar interactions is somewhat less suitable. Besides the contacts of the N-terminal amino group with Ala348 (CO, 1.99 Å) and Glu350 (COOH, 2.27 Å and 2.65 Å), only one specific hydrogen contact is identified, namely, between one of the phosphinate oxygen atoms and imidazole NH of His383 (2.90 Å). On the other hand, convenient face-to-face π-π stacking of the side chain aromatic rings of (*S*)-**6b** with the enzyme residues is visible. This stacking involves phenyl of (*S*)-**6b** and the aromatic ring of Phe467 in the S1 pocket, and 4-bromophenyl and imidazole of His383 in the S1′ pocket (Figure 3b).

**Figure 4:**
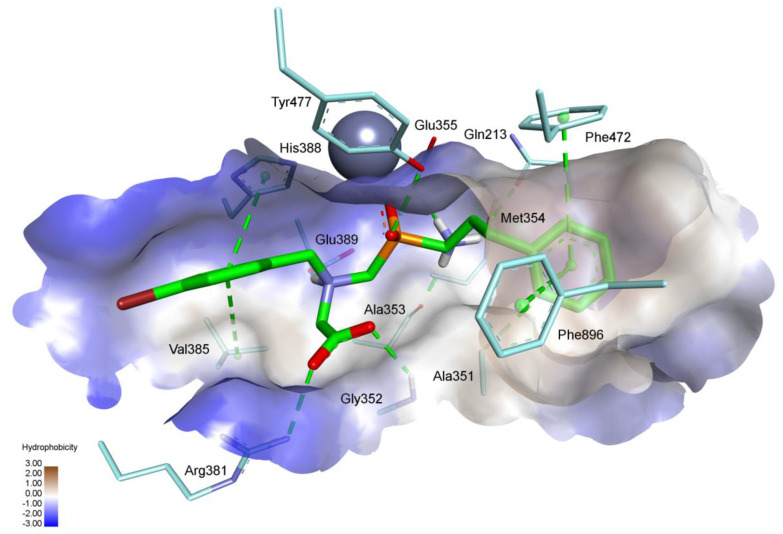
Modeled binding mode of (*R*) enantiomer of phosphinic dipeptide analog **6b** containing a 4-bromobenzyl P1′ fragment in the active center of human aminopeptidase N (PDB: 4FYT) [24]. Amino acid residues of the inhibitor and enzyme are shown as sticks, while the zinc ion is shown as a gray sphere. Hydrogen bonds and nonpolar interactions are shown as thin dashed lines, while the surface of the active site is colored according to hydrophobicity.

In accordance with its high potency, compound (*R*)-**6b** binds with *Hs*APN very tightly (Figure 4). Similarly to the complex of *Ss*APN, monodentate phosphinate-Zn coordination is associated with specific hydrogen bonding that involves the P-O and OH group of Tyr477 (2.59 Å). The N-terminal amino group is located in a polar environment, which results in a set of strong contacts with neighboring residues: Gln213 (CO, 2.60 Å), Ala353 (CO, 1.94 Å), Met354 (S, 2.58 Å), and Glu355 (COOH, 2.36 Å). C-Terminal carboxylate forms a salt bridge with the guanidino group of Arg381 (2.78 Å) and an H-bond with NH of Gly352 (2.43 Å). The protonated tertiary amino group delivers a component for another salt bridge with carboxylate of Glu389 (2.05 Å, which also corresponds with (*R*)-**6b**-*Ss*APN complex). Finally, the arylalkyl side chains are surrounded by hydrophobic residues. P1 phenylethyl is nested by Ala351, Phe472, and Phe896, while P1′ 4-bromobenzyl by Val385 and His388 (Figure 4).

**Figure 5:**
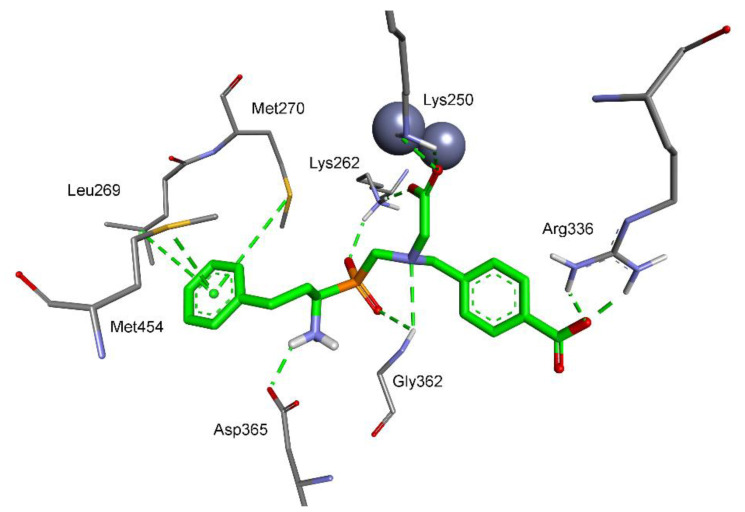
Modeled binding mode of phosphinic dipeptide analog (*R*)-**6c** containing a 4-carboxybenzyl P1′ fragment in the active center of bovine leucine aminopeptidase (PDB: 1LAM) [25]. Amino acid residues of the inhibitor and enzyme are shown as sticks, while zinc ions are shown as gray spheres. Hydrogen bonds and nonpolar interactions are shown as thin dashed lines.

Molecular modeling of the most active phosphinic compound **6c** with di-zinc leucine aminopeptidase (the crystal structure of bovine LAP was used [25]) revealed a non-typical mode of binding. C-terminal carboxylate, not phosphinate, is involved in coordination with the catalytic metal ions (Figure 5). The position of the carboxylate functionality is further stabilized by electrostatic contacts of both oxygen atoms with the amino groups of Lys250 (2.42 Å and 2.34 Å) and Lys262 (2.07 Å). The result of such a location is a shift of the whole molecule further to the S1 region. The oxygen atoms of the phosphinate moiety are involved in ionic interaction with NH_3_^+^ of Lys262 (2.08 Å) and hydrogen bonding with NH of Gly362 (2.68 Å). The N-terminal amino group forms a salt bridge with Asp365 (2.02 Å). The aromatic ring of the phenylethyl P1 residue is in a hydrophobic environment provided by the side chains of Leu269, Met270, and Met454. The P1′ aromatic fragment is not so tightly inserted into the S1′ pocket; nevertheless, the phenyl-attached carboxylate group holds the position typical for the C-termini of native substrates, and forms specific interactions with the guanidine group of Arg336 (2.02 Å and 1.87 Å).

**Figure 6:**
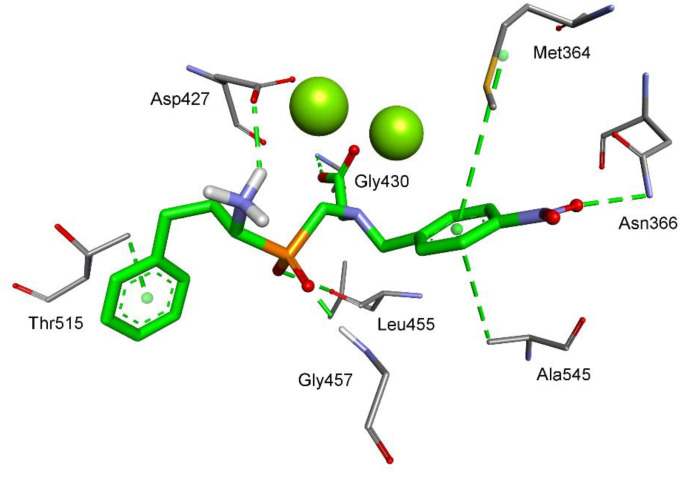
Modeled binding mode of phosphinic dipeptide analog (*R*)-**6d** containing a 4-nitrobenzyl P1′ fragment in the active center of tomato leucine aminopeptidase (PDB: 4KSI) [26]. Amino acid residues of the inhibitor and enzyme are shown as sticks, while magnesium ions are shown as green spheres. Hydrogen bonds and nonpolar interactions are shown as thin dashed lines.

As barley leucine aminopeptidase was not crystallized, the structure of tomato LAP (the only plant resolved) was used for molecular modeling [26]. The structure contains two magnesium ions in the active center. Similarly to *Ss*LAP, the most active phosphinic compound **6d** coordinates the metallic site with the C-terminal carboxylate functionality (Figure 6). One of the carboxylate oxygen atoms is also involved in the hydrogen bond with NH of Gly430 (2.19 Å). Significantly, the overall conformation of the ligand is reversed. Phenylethyl, the nominal P1 residue, is docked in the S1′ binding pocket, and vice versa, the P1′ 4-nitrobenzyl side chain is located in the S1 subsite. Interactions of the latter are particularly favorable: The phenyl ring is positioned between the side chains of Met364 and Ala545, while the nitro group is pointed at NH of Asn366 (3.08 Å). The other functionalities of the molecule are also conveniently bound. Both phosphinate oxygen atoms are expected to be involved in hydrogen bonding, specifically with CO of Leu455 (2.97 Å) and NH of Gly457 (1.70 Å). The N-terminal amino group forms a salt bridge with Asp427 (2.21 Å).

### 2.4. Summary

We proposed a simple method for preparation of pseudodipeptide analogs, which included N-substituted aminomethylphosphinic structural motif, -P(O)(OH)-CH_2_-NH(R)-, introduced instead of the core amide bond. The compounds were obtained in three-component Mannich-type condensation of α-aminoalkyl-*H*-phosphinic acid (P-H component), formaldehyde (carbonyl component), and N-substituted glycines (amino component). Inspired by the biological activity potential of target compounds, we used the amino acid substrates of dedicated structures, namely, the analog of homophenylalanine as the H-phosphinic component, and *N*-benzylglycines as the amino components. These precursors were readily available. Carrying out the whole procedure appeared fairly convenient, although not high yielding (not optimized). Intermediates and final compounds were obtained as satisfactory pure solids crystallizing after the reactions, and they did not demand any further purification steps.

The approach can be recommended for the synthesis of multifunctional compounds of diverse applications. In the current work, we verified the suitability of extension of the pseudipeptide backbone and transfer of the P1′ substituent from the α carbon to the internal amino group for construction of metalloaminopeptidase inhibitors. The resulting phosphinates were found to be moderate inhibitors of mammalian and plant alanyl and leucine metalloaminopeptidases. For human APN, potent submicromolar activity was measured with halogen- and nitro-substituted compounds **6a**, **6b**, and **6d**. It can be concluded that the extended pseudodipeptide analogs demand large and hydrophobic catalytic sites to be well accepted. Indeed, only for APNs the modeled ligand–protein complexes revealed the binding modes similar to that presumed for the transition states of hydrolysis of natural substrates. The docking data also emphasized the role of the protonation of the internal amino group, which formed specific hydrogen bonds when ionized. For LAPs, with optimal activity at a higher pH, this group was presumably not protonated and interacted not so effectively. Binding modes were found distorted, in particular, the phosphinate group was not involved in coordination of the catalytic metal ions. The chelation role was taken over by the C-terminal carboxylate functionality, which fundamentally changed the overall conformations.

## 3. Materials and Methods

### 3.1. General

Chemical and biochemical materials were purchased from Sigma-Aldrich (Poznań, Poland) and Avantor Performance Materials (Gliwice, Poland), and were used without further purification. Fluorogenic substrate Ala-AMC (l-alanine-4-methylcoumaryl-7-amide) was purchased from PeptaNova (Sandhausen, Germany). Microsomal alanyl aminopeptidase from porcine kidney (*Ss*APN) was purchased from Sigma-Aldrich (Poznań, Poland), and recombinant human alanine aminopeptidase (*Hs*APN) from R&D System (Minneapolis, MN, USA). Leucine aminopeptidase from porcine kidney (*Ss*LAP) [26] and barley seeds (*Hv*LAP) [21] were isolated and purified according to the procedures described previously. ^1^H, ^13^C, and ^31^P NMR spectra were recorded on a 400YH (JEOL, Tokyo, Japan) spectrometer at 295 K. Mass spectra analyses were performed on a spectrometer LC-MS QDa (Waters e2695, 2489 UV/Vis Detector, Acquity QDa detector). HRMS spectra were registered using a Waters LCT Premier XE mass spectrometer (electrospray ionization, ESI) (Waters, Milford, MA, USA). The progress of the enzymatic reactions was monitored spectrophotometrically (UV-VIS Spectrophotometer, JASCO V-730) by following the change in absorbance at 405 nm (formation of p-nitroaniline), or by measuring fluorescence with a spectrofluorometer SpectraMax Gemini EM (Molecular Devices, San Jose, CA, USA) operating at two wavelengths: excitation 355 nm and emission 460 nm (for AMC).

### 3.2. Synthetic Procedure

*1-(N-Benzyloxycarbonylamino) 3-phenylpropyl-H-phosphinic acid***2** [5,13] (1.0 g, 3.0 mmol) and an *N-benzylated amino acid* (**4a**–**f**) [14] (3.0 mmol) were dissolved in a hot water/acetic acid/concentrated hydrochloric acid mixture (10/10/0.25 mL). Formaldehyde (0.46 mL of 36–38% aqueous solution, 0.5 g, 6.0 mmol, 2.0 eq.) was added and the mixture was refluxed for 5 h. After cooling, the solution was left in a refrigerator for crystallization (for one to several days). The precipitated solid (**5a**–**d**) was filtered, washed with diethyl ether and dried in the air. The Cbz was removed in HBr (33% solution in AcOH, 10 mL per 1 g) by stirring 2 h at room temperature. The acids were removed under reduced pressure and the residue was dissolved in ethanol (10–20 mL) and neutralized with propylene oxide. The solvent was removed under reduced pressure and the residue triturated with diethyl ether. The resulting white solid (**6a**–**d**) was filtered, washed with diethyl ether, and dried in the air.

*N-{[1-Amino-3-phenylpropyl(hydroxy)phosphoryl]methyl}-N-2,4-dichlorobenzylaminoacetic acid* (**6a**). Yield: 41%, condensation, and 92%, N-deprotection. ^1^H NMR (400 MHz, D_2_O + NaOD) δ: 7.28–7.06 (m, 8H, 3H_ar_ + Ph); 3.66 (AB system, *J* = 14.0 Hz, 2H, NCH_2_CO); 3.16 (AB system, *J* = 16.4 Hz, 2H, NCH_2_C_ar_); 2.78 (m, 1H, PCH_2_N); 2.64 (m, 2H, CH + PCH_2_N); 2.44 (m, 2H, CH_2_Ph); 1.70 and 1.30 (m and m, 1H and 1H, CH_2_). ^31^P NMR (162 MHz, D_2_O + NaOD) δ: 40.59. ^13^C NMR (101 MHz, D_2_O + NaOD) δ: 179.57, 142.22, 135.22, 134.73, 132.94, 132.27, 129.03, 128.68, 128.60, 127.19, 126.04, 58.86 (d, *J* = 6.1 Hz), 56.18 (d, *J* = 8.1 Hz), 51.23 (d, *J* = 103.2 Hz), 49.07 (d, *J* = 99.0 Hz), 32.12 (d, *J* = 12.1 Hz), 31.28. MS calcd for C_19_H_23_Cl_2_N_2_O_4_P: 444.08, found 443.07 [M − H]. HRMS calcd for C_19_H_23_Cl_2_N_2_O_4_P: 444.0772, found 445.0852 [M + H].

*N-{[1-Amino-3-phenylpropyl(hydroxy)phosphoryl]methyl}-N-4-bromobenzylaminoacetic acid* (**6b**). Yield: 40%, condensation, and 95%, N-deprotection. ^1^H NMR (400 MHz, D_2_O + NaOD) δ: 7.37 (d, *J* = 8.1 Hz, 2H, 2H_ar_); 7.23 (m, 2H, 2H_ar_); 7.14 (m, 5H, Ph); 3.52 (AB system, *J* = 13.2 Hz, 2H, NCH_2_CO); 3.08 (AB system, *J* = 16.3 Hz, 2H, NCH_2_C_ar_); 2.71 (m, 2H, CH + PCHN); 2.50 (m, 3H, PCHN + CH_2_Ph); 1.76 and 1.40 (m and m, 1H and 1H, CH_2_). ^31^P NMR (162 MHz, D_2_O + NaOD) δ: 40.58. ^13^C NMR (101 MHz, D_2_O + NaOD) δ: 179.55, 142.36, 137.92, 131.46, 131.39, 128.77, 128.71, 126.12, 120.57, 59.27 (d, *J* = 6.1 Hz), 58.92 (d, *J* = 8.1 Hz), 51.30 (d, *J* = 103.0 Hz), 49.08 (d, *J* = 96.0 Hz), 32.13 (d, *J* = 12.1 Hz), 31.37. MS calcd for C_19_H_24_BrN_2_O_4_P: 454.07, found 453.04 [M − H]. HRMS calcd for C_19_H_24_BrN_2_O_4_P: 454.0657, found 455.0739 [M + H].

*N-{[1-Amino-3-phenylpropyl(hydroxy)phosphoryl]methyl}-N-4-carboxybenzylaminoacetic acid* (**6c**). Yield: 43%, condensation, and 97%, N-deprotection. ^1^H NMR (400 MHz, D_2_O + NaOD) δ: 7.69 (d, *J* = 7.9 Hz, 2H, 2H_ar_); 7.27 (d, *J* = 7.9 Hz, 2H, 2H_ar_); 7.19 (m, 2H, 2H_ar_); 7.10 (m, 3H, 3H_ar_); 3.60 (AB system, *J* = 13.3 Hz, 2H, NCH_2_CO); 3.10 (AB system, *J* = 16.3 Hz, 2H, NCH_2_C_ar_); 2.71 (m, 2H, CH + PCHN); 2.56–2.40 (m, 3H, PCHN + CH_2_Ph); 1.74 and 1.37 (m and m, 1H and 1H, CH_2_). ^31^P NMR (162 MHz, D_2_O + NaOD) δ: 40.66. ^13^C NMR (101 MHz, D_2_O + NaOD) δ: 179.60, 175.47, 142.33, 142.00, 135.21, 129.36, 129.04, 128.70, 128.66, 126.10, 59.36 (d, *J* = 7.1 Hz), 59.28 (d, *J* = 9.1 Hz), 51.37 (d, *J* = 104.0 Hz), 49.08 (d, *J* = 96.0 Hz), 32.13 (d, *J* = 12.1 Hz), 31.38. MS calcd for C_20_H_25_N_2_O_6_P: 420.14, found 419.15 [M − H]. HRMS calcd for C_20_H_25_N_2_O_6_P: 420.1450, found 421.1530 [M + H].

*N-{[1-Amino-3-phenylpropyl(hydroxy)phosphoryl]methyl}-N-4-nitrobenzylaminoacetic acid* (**6d**). Yield: 36%, condensation, and 96%, N-deprotection. ^1^H NMR (400 MHz, D_2_O + NaOD) δ: 7.96 (d, *J* = 8.8 Hz, 2H, 2H_ar_); 7.37 (d, *J* = 8.8 Hz, 2H, 2H_ar_); 7.13 (m, 2H, 2H_ar_); 7.04 (m, 3H, 3H_ar_); 3.63 (AB system, *J* = 14.0 Hz, 2H, NCH_2_CO); 3.07 (AB system, *J* = 16.3 Hz, 2H, NCH_2_C_ar_); 2.73–2.62 (m, 2H, CH + PCHN); 2.52–2.38 (m, 3H, PCHN + CH_2_Ph); 1.68 and 1.31 (m and m, 1H and 1H, CH_2_). ^31^P NMR (162 MHz, D_2_O + NaOD) δ: 40.38. ^13^C NMR (101 MHz, D_2_O + NaOD) δ: 179.36, 147.27, 146.62, 142.13, 130.07, 128.58, 128.49, 126.00, 123.61, 59.36 (d, *J* = 7.1 Hz), 58.95 (d, *J* = 8.1 Hz), 51.53 (d, *J* = 103.0 Hz), 49.06 (d, *J* = 96.0 Hz), 31.99 (d, *J* = 12.1 Hz), 31.21. MS calcd for C_19_H_24_N_3_O_6_P: 421.14, found 420.13 [M − H]. HRMS calcd for C_19_H_24_N_3_O_6_P: 421.1403, found 422.1489 [M + H].

*N-{[1-Amino-3-phenylpropyl(hydroxy)phosphoryl]methyl}aminoacetic acid* (**6g**). Compound **5d** (0.56 g, 1.0 mmol) was refluxed with SnCl_2_ (0.95 g, 5.0 mmol, 5.0 eq.) in 15 mL of ethanol for 3 h. After cooling, the pH of the solution was adjusted at 9–10 with 10% NaHCO_3_. The precipitated solid was filtered on celite. The filtrate was concentrated under reduced pressure and acidified to pH = 4–5 with 1 M HCl. The precipitated dipeptide was filtered, washed with water, and dried in the air. N-Cbz removal was performed according to the general procedure described above. The overall yield: 46%. ^1^H NMR (400 MHz, D_2_O + NaOD) δ: 7.21 (m, 4H, 4H_ar_); 7.12 (m, 1H, H_ar_); 3.03 (s, 2H, NCH_2_CO); 2.78 (m, 1H, CH); 2.66 (m, 2H, PCH_2_N); 2.51 (m, 2H, CH_2_Ph); 1.89 and 1.54 (m and m, 1H and 1H, CH_2_). ^31^P NMR (162 MHz, D_2_O + NaOD) δ: 40.29. ^13^C NMR (101 MHz, D_2_O + NaOD) δ: 192.25, 142.39, 128.77, 128.67, 126.17, 54.05 (d, *J* = 13.5 Hz), 48.78 (d, *J* = 98.7 Hz), 45.69 (d, *J* = 96.3 Hz), 32.23 (d, *J* = 11.7 Hz), 32.10. MS calcd for C_12_H_19_N_2_O_4_P: 286.11, found 285.07 [M − H]. HRMS calcd for C_12_H_19_N_2_O_4_P: 286.1082, found 287.1166 [M + H].

### 3.3. Inhibition Studies

The current studies followed the methodologies of the enzyme kinetic experiments, with calculation of the inhibition constants, and the mechanism of inhibition, which are presented in detail in our previous papers: For microsomal alanyl aminopeptidase from porcine kidney [27,28,29], recombinant human alanine aminopeptidase [30], leucine aminopeptidase from porcine kidney [27,28,31], and leucine aminopeptidase from barley seeds (*Hv*APN) [22,28,29].

### 3.4. Modeling

Crystal structures of following enzymes were obtained from the Research Collaboratory for Structural Bioinformatics Protein Data Bank (RCSB-PDB): porcine (*Sus scrofa*) alanyl M1 aminopeptidase, PDB: 4FKE [23], human (*Homo sapiens*) M1 aminopeptidase, PDB: 4FYT [24], bovine (*Bos taurus*) M17 leucine aminopeptidase PDB: 1LAM [25], and tomato (*Solanum lycopersicum*) M17 acidic leucine aminopeptidase PDB: 4KSI [26]. Using options implemented in Protein Preparation Wizard, the structures were prepared for calculation: all of the water molecules and the ligands were removed, and the proteins were protonated at the experimental pH [32]. LigPrep [33] was used to create the stereoisomers of the final compounds and to pronate them at the experimental pH. Epik and OPLS3e force fields were used for the geometry optimization, and the metal binding states were added to the calculations [32]. The penultimate stage was induced fit docking (IFD) [34]. All of the stereomeric compounds were docked in the grid created for the selected enzymes. The box center was set on the center of the metal or metals with size of 20 Å. The VSGB (variable-dielectric generalized born) model, which incorporates residue-dependent effects, was used. The solvent was water. The side chains were optimized within 5.0 Å of ligand poses, and Glide redocking was carried out with the XP (extra precision) algorithm. The top pose for each ligand was saved.

## Figures and Tables

**Table 1:** Activity of pseudodipeptides as inhibitors of aminopeptidases: porcine and human APNs, and porcine and barley seeds LAPs (the most significant inhibition is indicated in bold, NT—not tested).

**Entry**	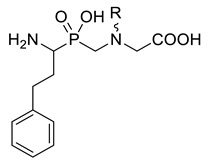	***K*_i_ [μM]**			
		***Ss*APN**	***Hs*APN**	***Ss*LAP**	***Hw*LAP**
**1**	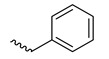	3.0 [7]	NT	52 [7]	NT
**6a**	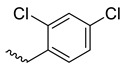	7.7	**0.10**	107	37
**6b**	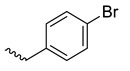	5.8	**0.13**	168	94
**6c**	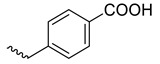	34	1.4	27 ^a^	36
**6d**	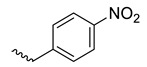	17	**0.24**	53 ^a^	13
**6g**	H	63	NT	29	173

^a^ Slow-binding inhibition, mechanism B.

## Supplementary Materials

The following are available online, Figures S1–S20: ^1^H, ^31^P and ^13^C NMR, and MS spectra of compounds **6a**–**d** and **6g**.
