# Supplementary material for: N-Benzyl Residues as the P1′ Substituents in Phosphorus-Containing Extended Transition State Analog Inhibitors of Metalloaminopeptidases
Source: Molecules. 2020 Sep 22;25(18):4334. doi: 10.3390/molecules25184334 (PMC7571175; doi:10.3390/molecules25184334)
Supplement: Supplementary file 1 [file molecules-25-04334-s001.pdf]

Supplementary Materials

for

N-Benzyl residues as the P1' substituents in phosphorus-containing *extended* transition state analog inhibitors of metalloaminopeptidases

Kamila Janiszewska, Michał Talma, Bartosz Oszywa, Małgorzata Pawełczak, Paweł Kafarski and Artur Mucha

Table of Contents

^{1}H NMR spectrum of compound **6a** 2
^{31}P NMR spectrum of compound **6a** 2
^{13}C NMR spectrum of compound **6a** 3
MS spectrum of compound **6a** [M-H] 3
^{1}H NMR spectrum of compound **6b** 4
^{31}P NMR spectrum of compound **6b** 4
^{13}C NMR spectrum of compound **6b** 5
MS spectrum of compound **6b** [M-H] 5
^{1}H NMR spectrum of compound **6c** 6
^{31}P NMR spectrum of compound **6c** 6
^{13}C NMR spectrum of compound **6c** 7
MS spectrum of compound **6c** [M-H] 7
^{1}H NMR spectrum of compound **6d** 8
^{31}P NMR spectrum of compound **6d** 8
^{13}C NMR spectrum of compound **6d** 9
MS spectrum of compound **6d** [M-H] 9
^{1}H NMR spectrum of compound **6g** 10
^{31}P NMR spectrum of compound **6g** 10
^{13}C NMR spectrum of compound **6g** 11
MS spectrum of compound **6g** [M-H] 11

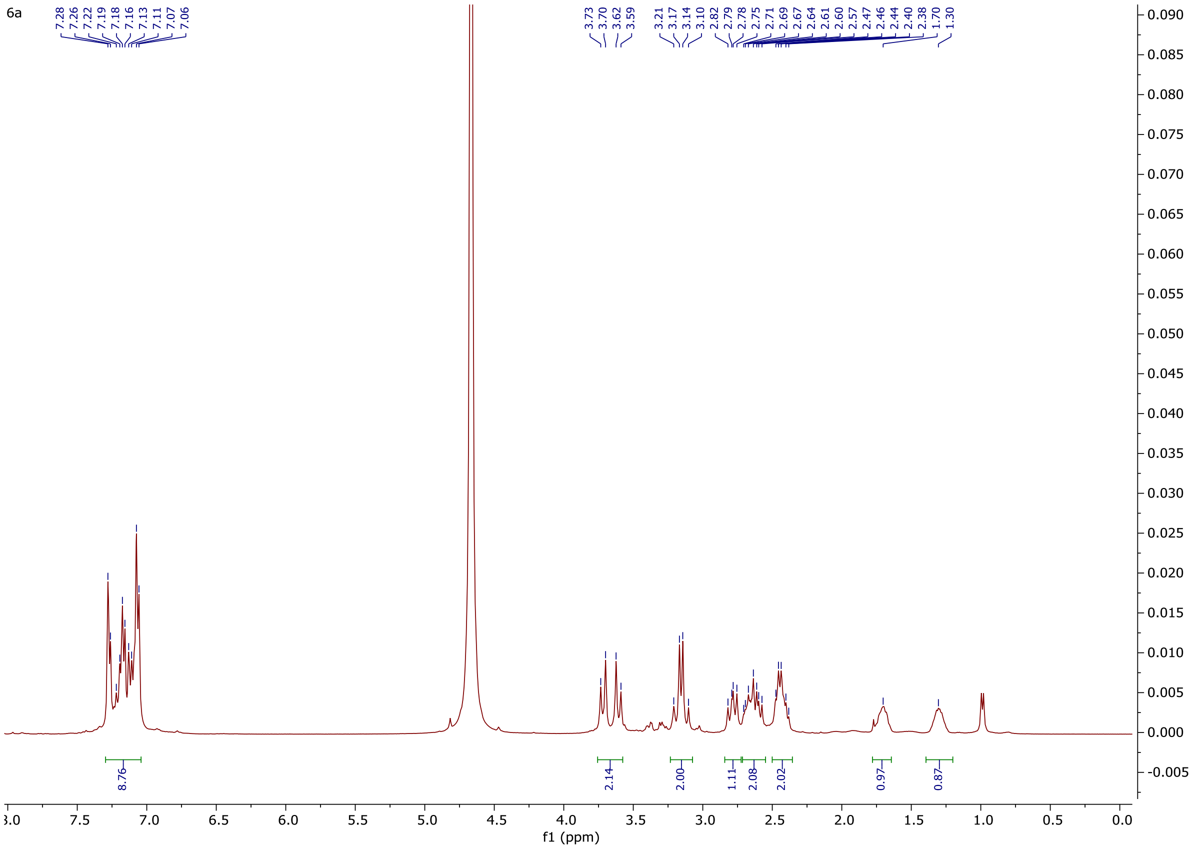

Figure S1. ^{1}H NMR spectrum of compound **6a**

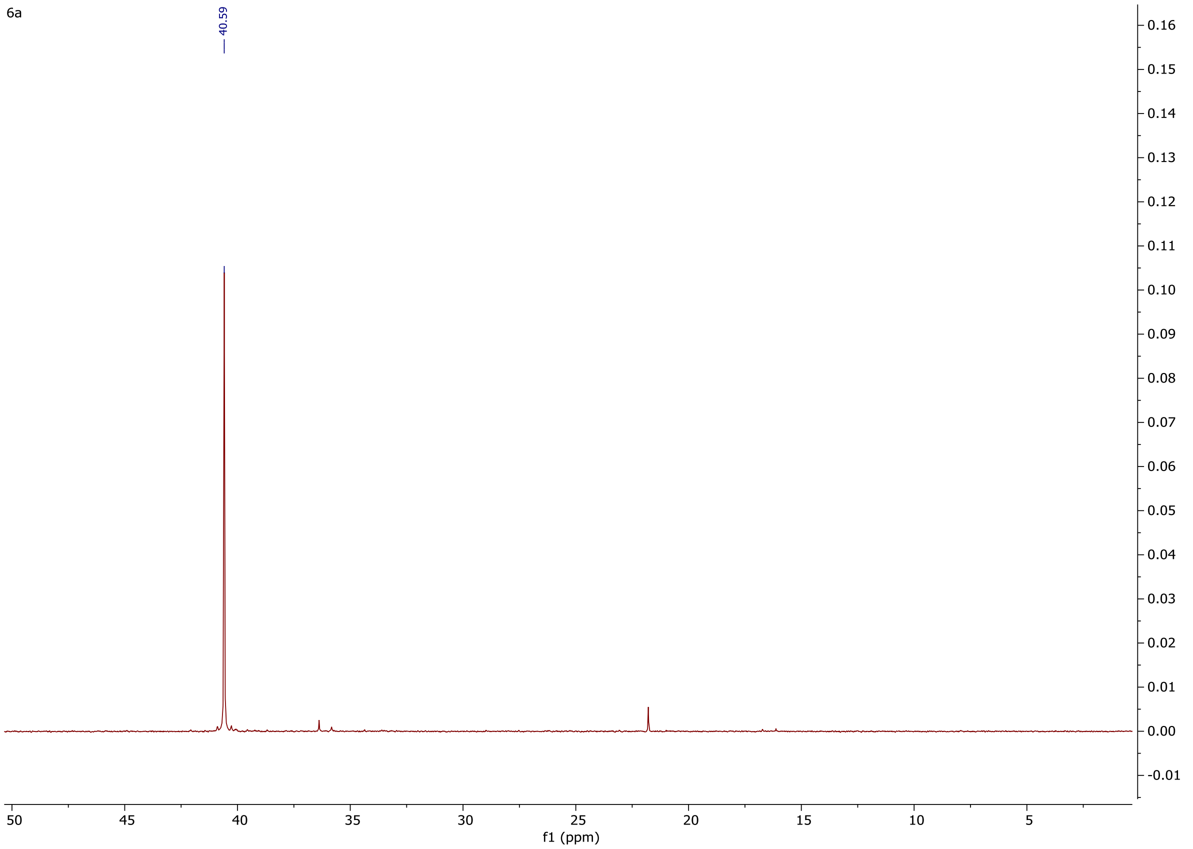

Figure S2. ^{31}P NMR spectrum of compound **6a**

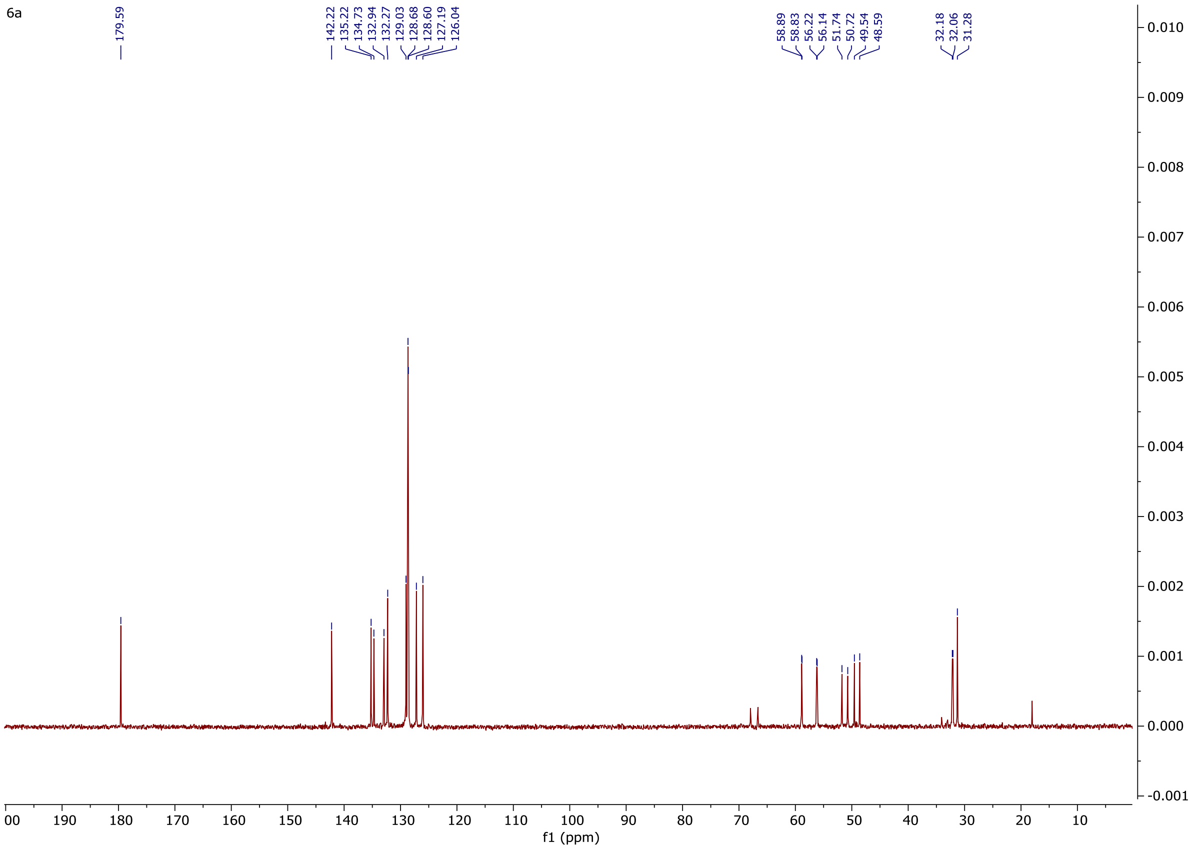

Figure S3. ^{13}C NMR spectrum of compound **6a**

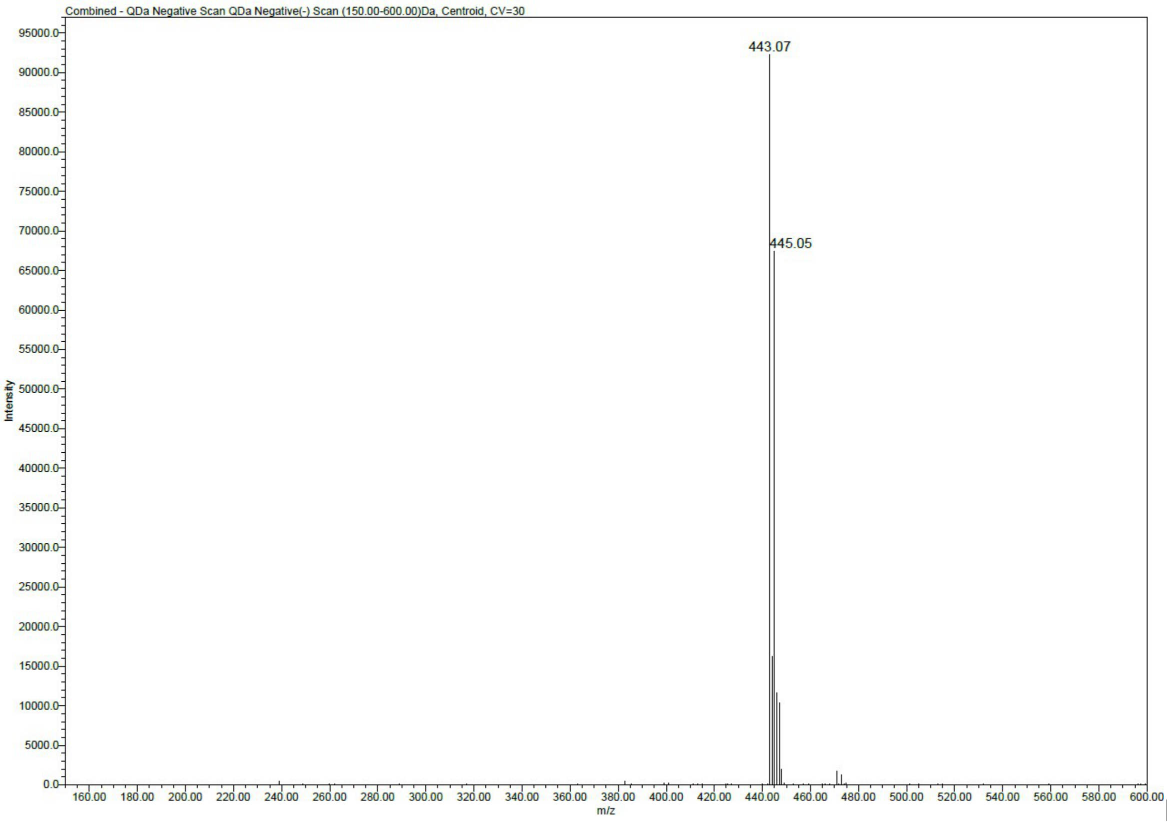

MS

Figure S4. MS spectrum of compound **6a** [M-H]

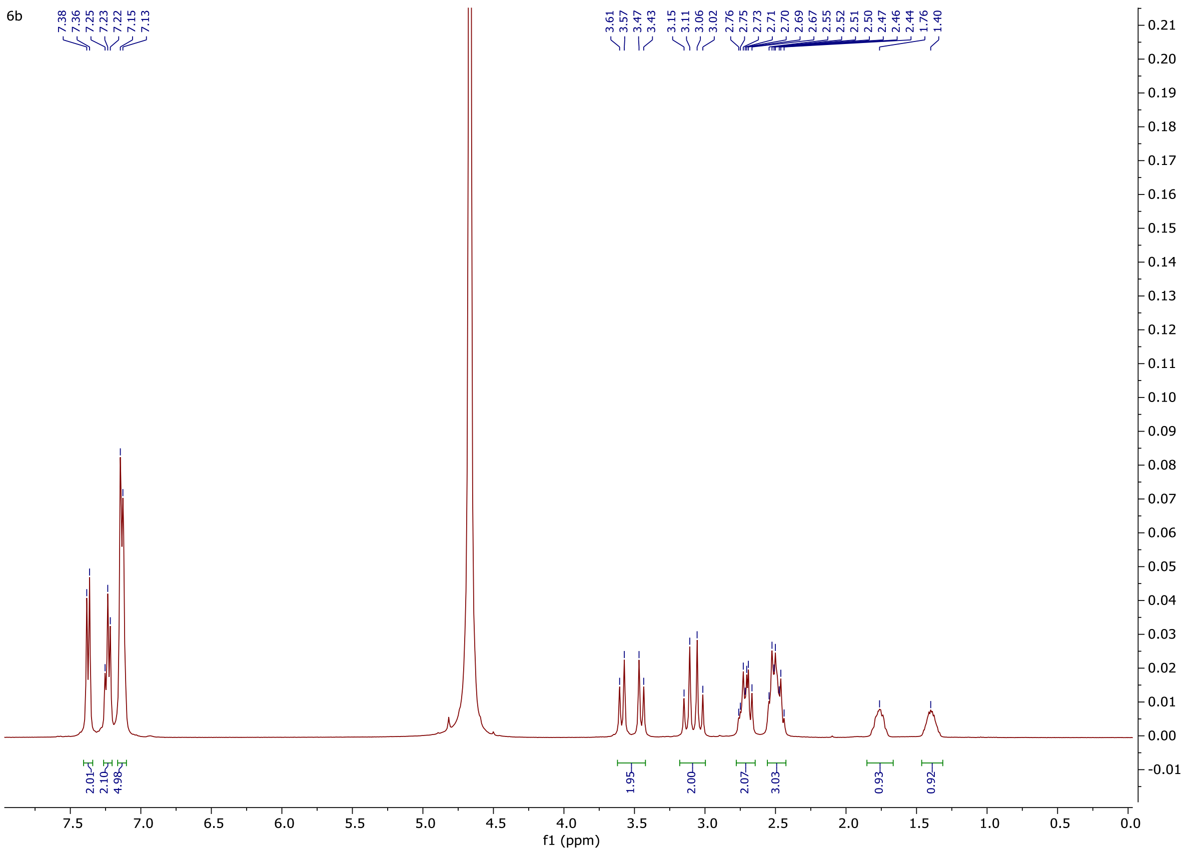

Figure S5. ^{1}H NMR spectrum of compound **6b**

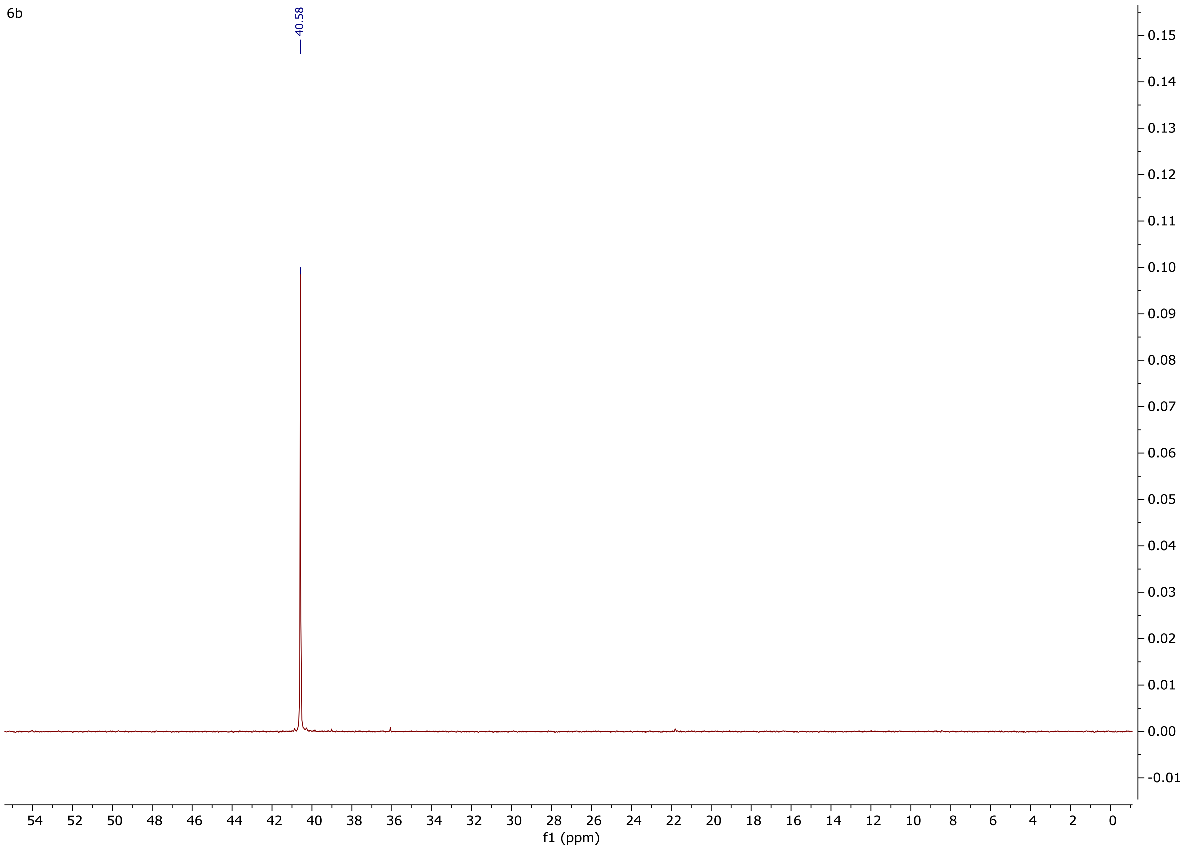

Figure S6. ^{31}P NMR spectrum of compound **6b**

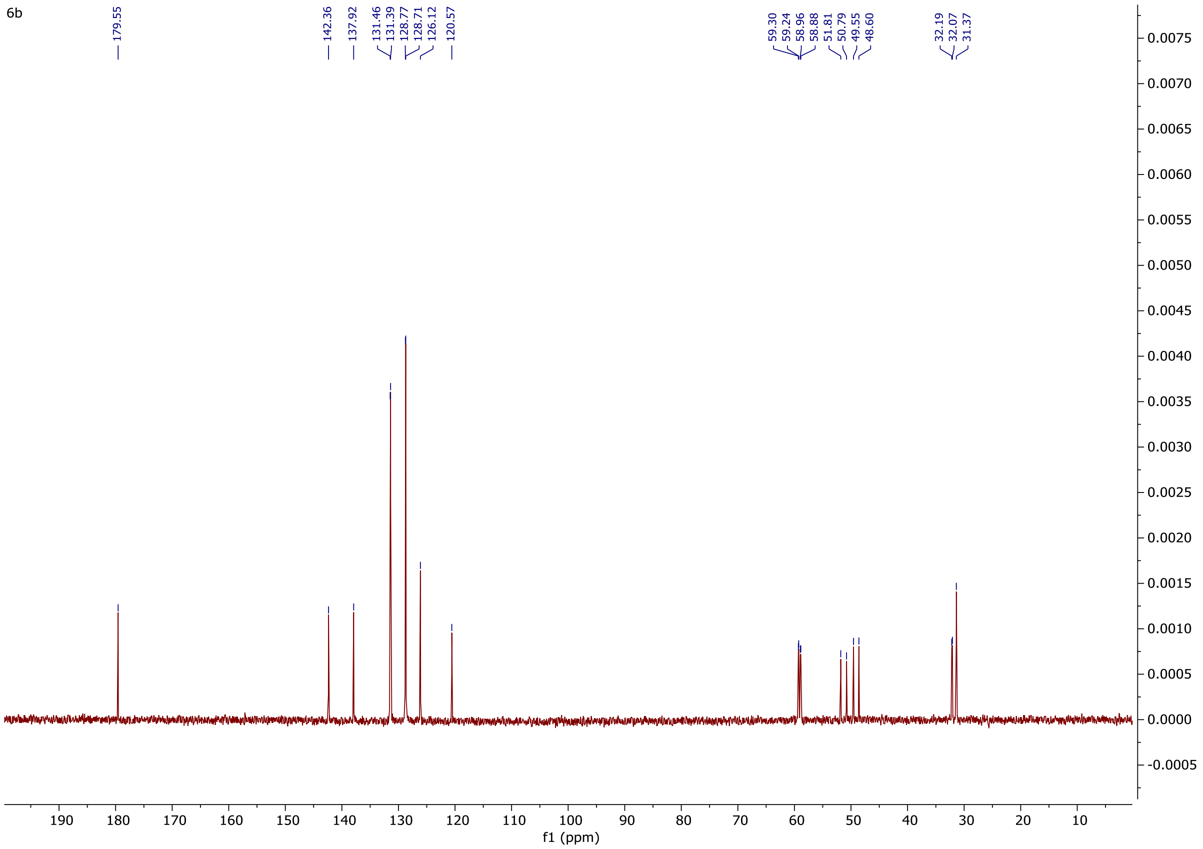

Figure S7. ^{13}C NMR spectrum of compound **6b**

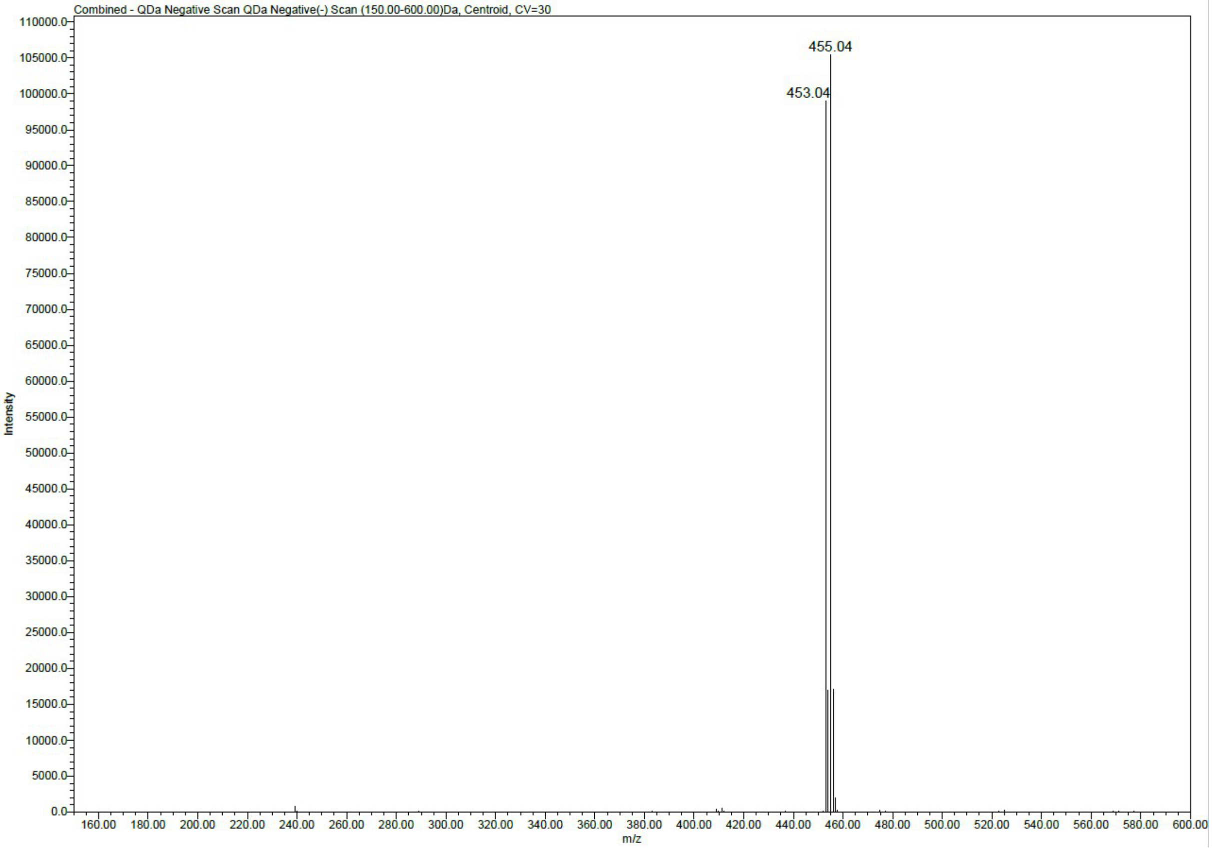

Figure S8. MS spectrum of compound **6b** [M-H]

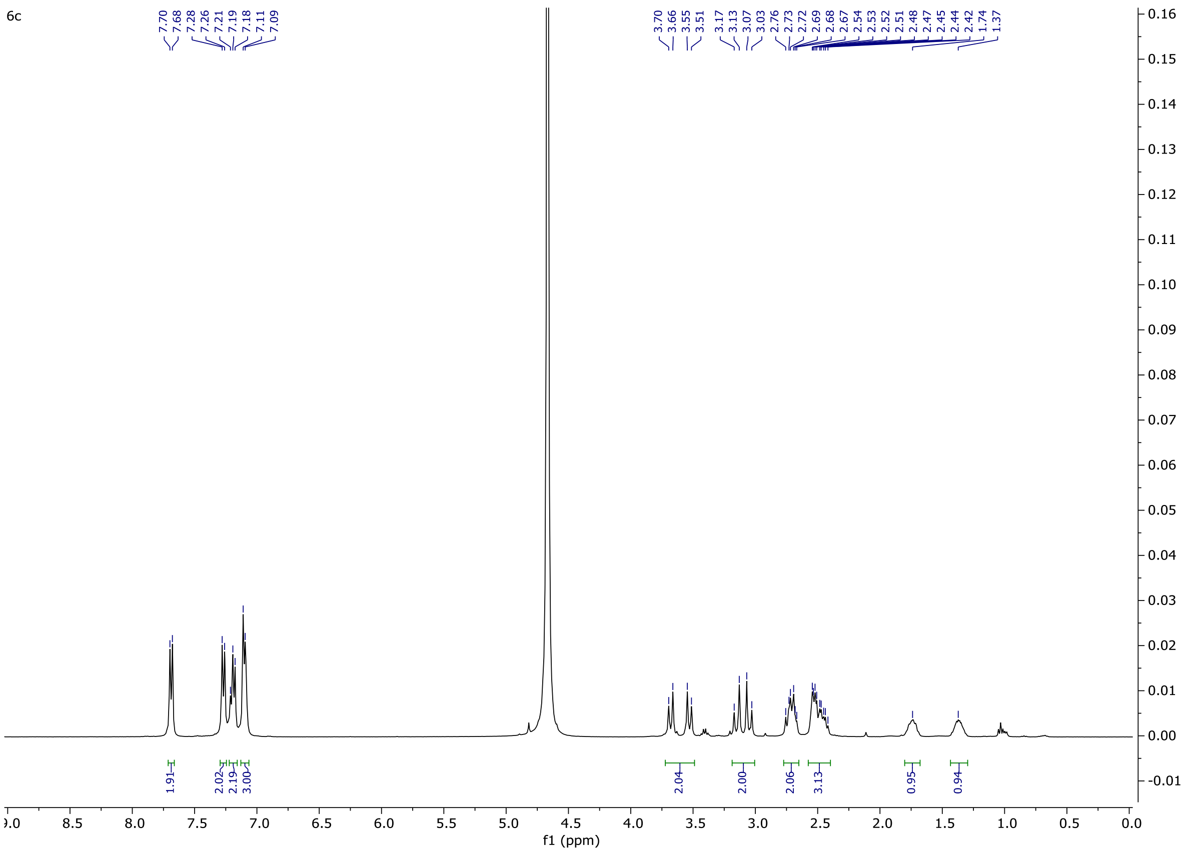

Figure S9. ^1H NMR spectrum of compound **6c**

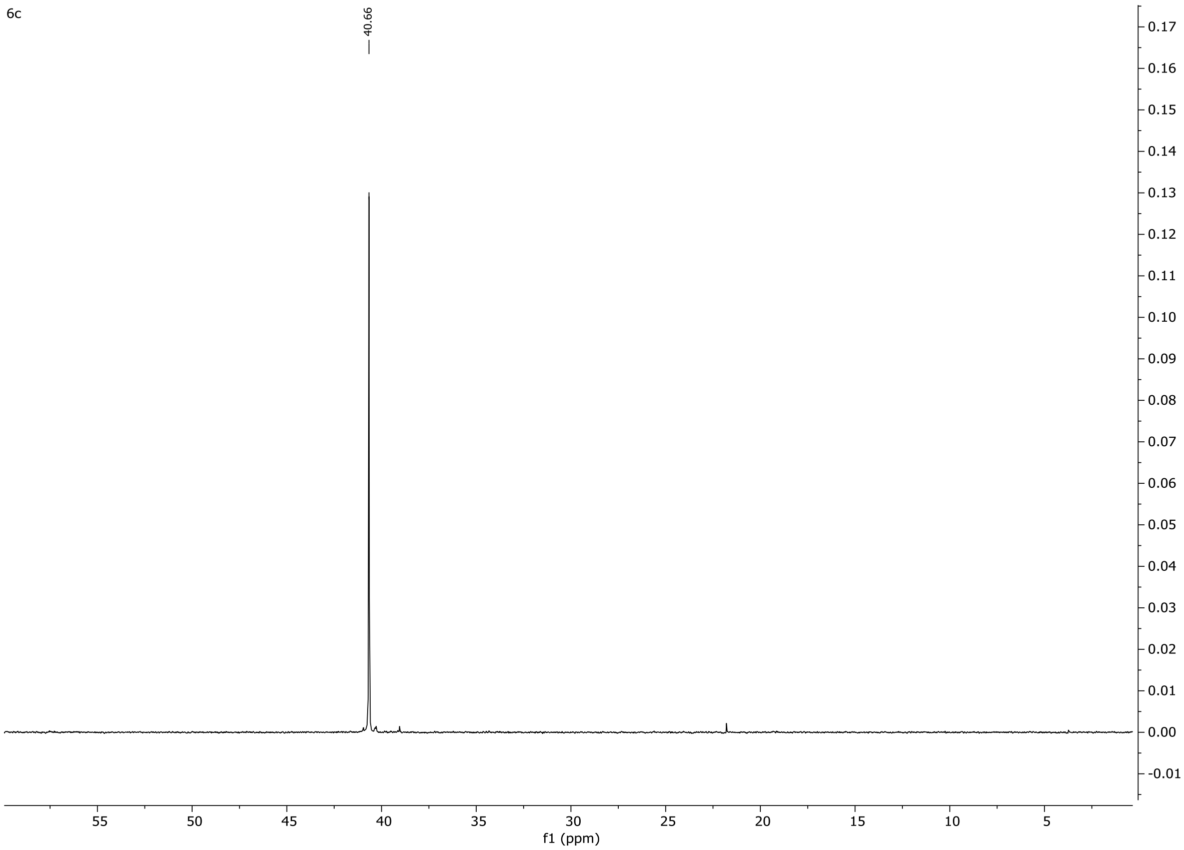

Figure S10. ^{31}P NMR spectrum of compound **6c**

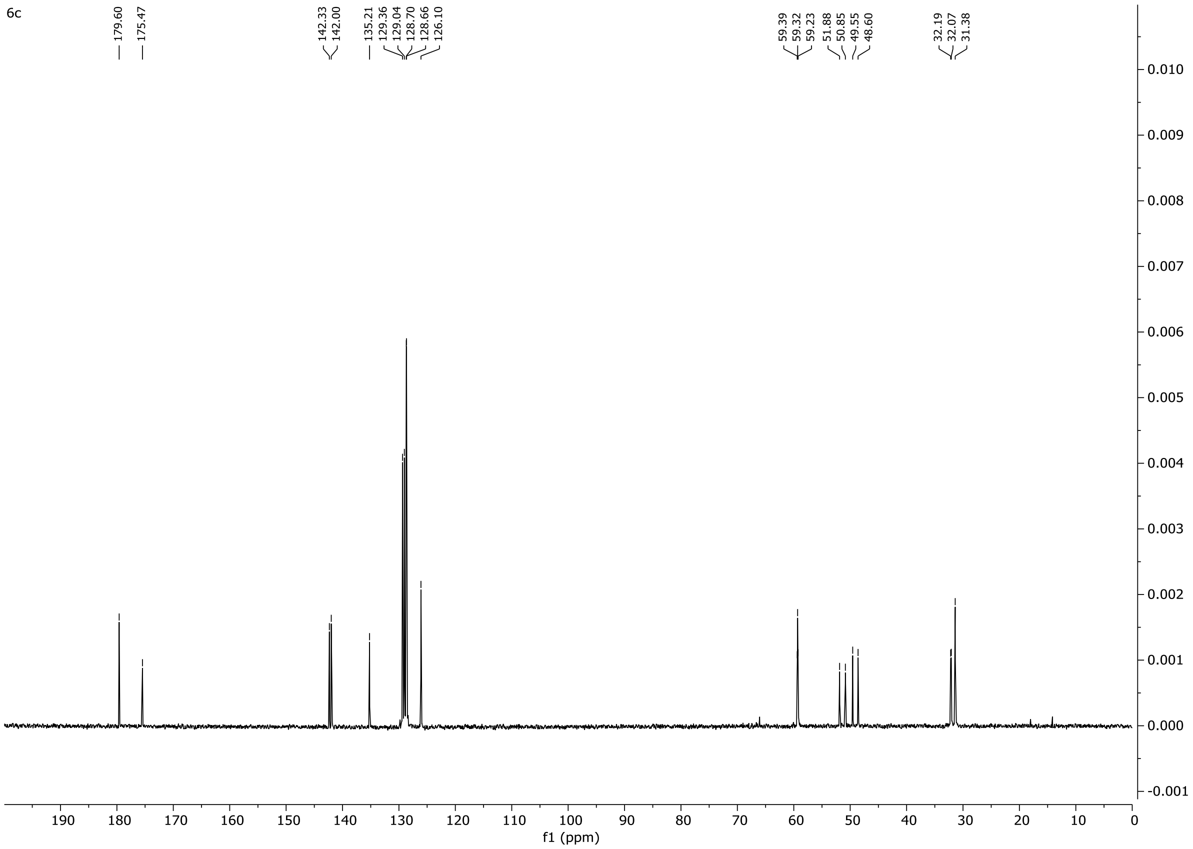

Figure S11. ^{13}C NMR spectrum of compound **6c**

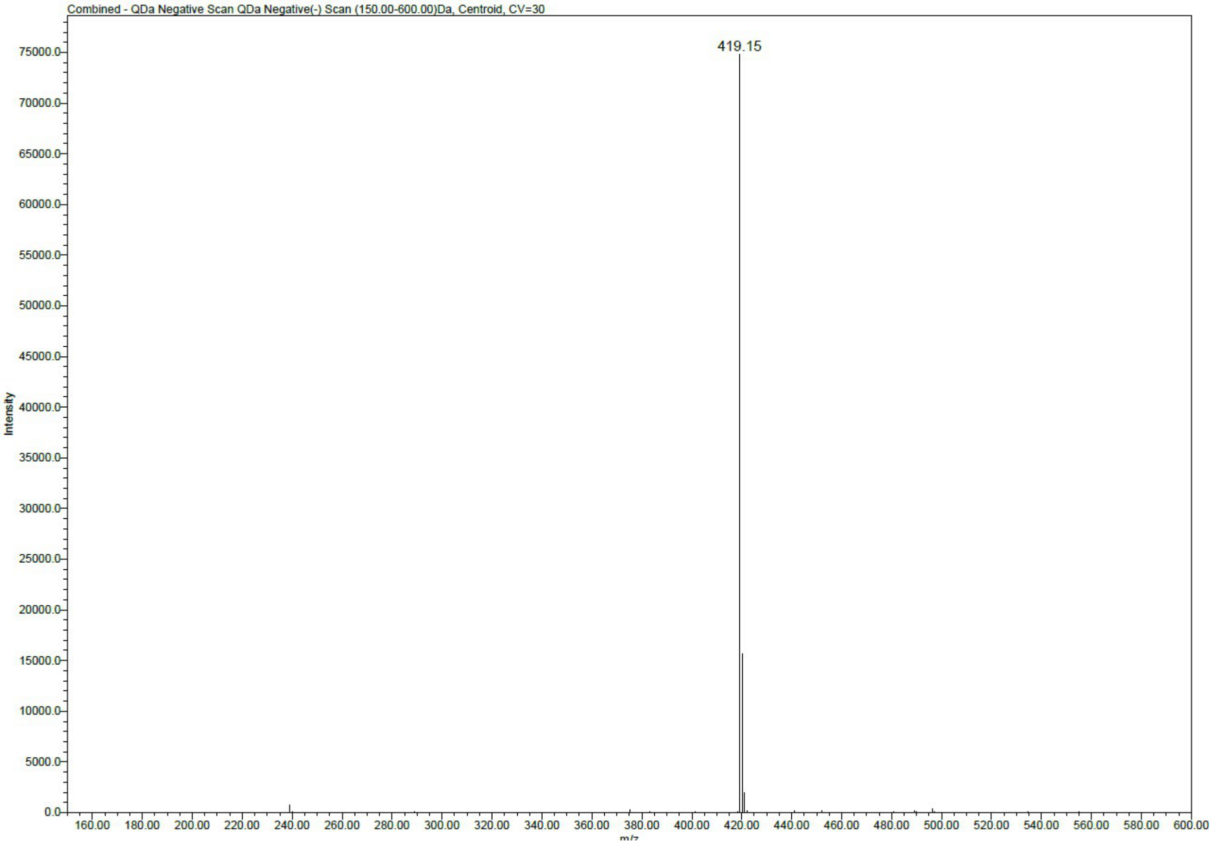

Figure S12. MS spectrum of compound **6c** [M-H]

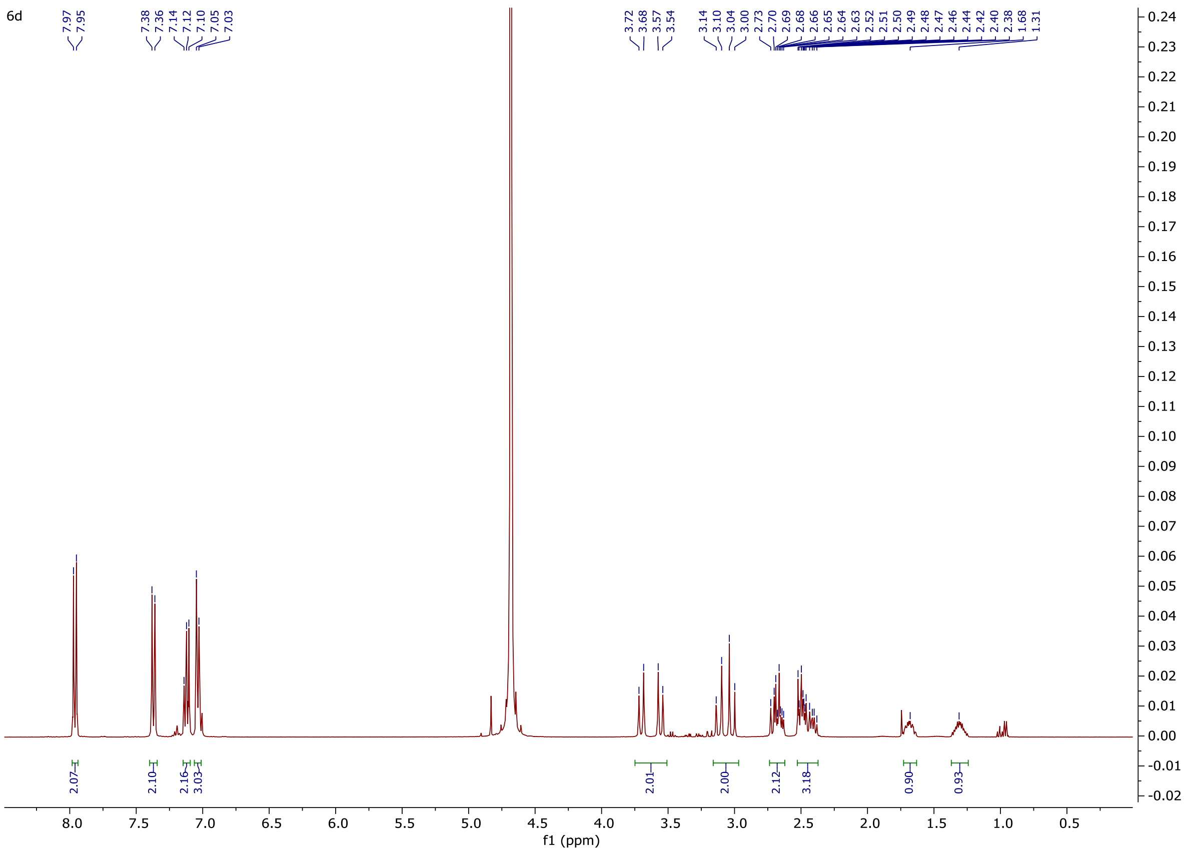

Figure S13. ^{1}H NMR spectrum of compound **6d**

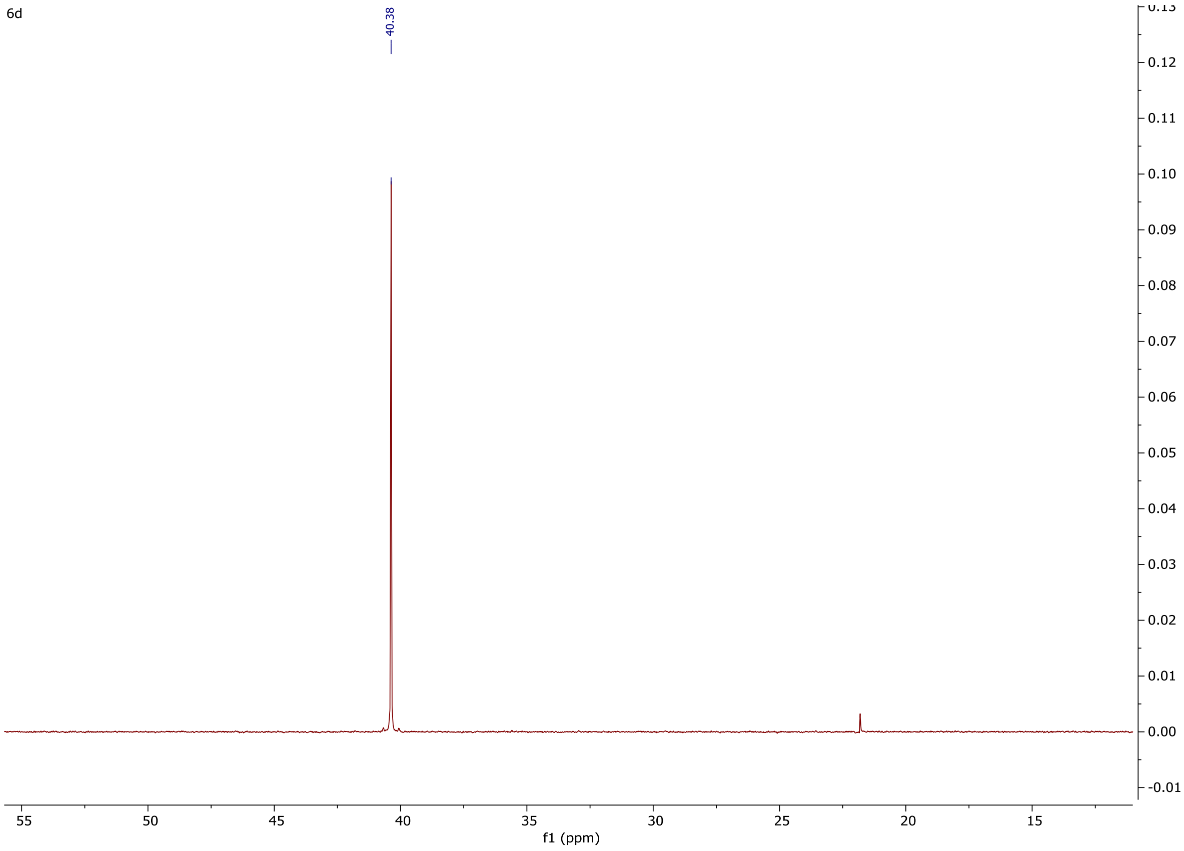

Figure S14. ^{31}P NMR spectrum of compound **6d**

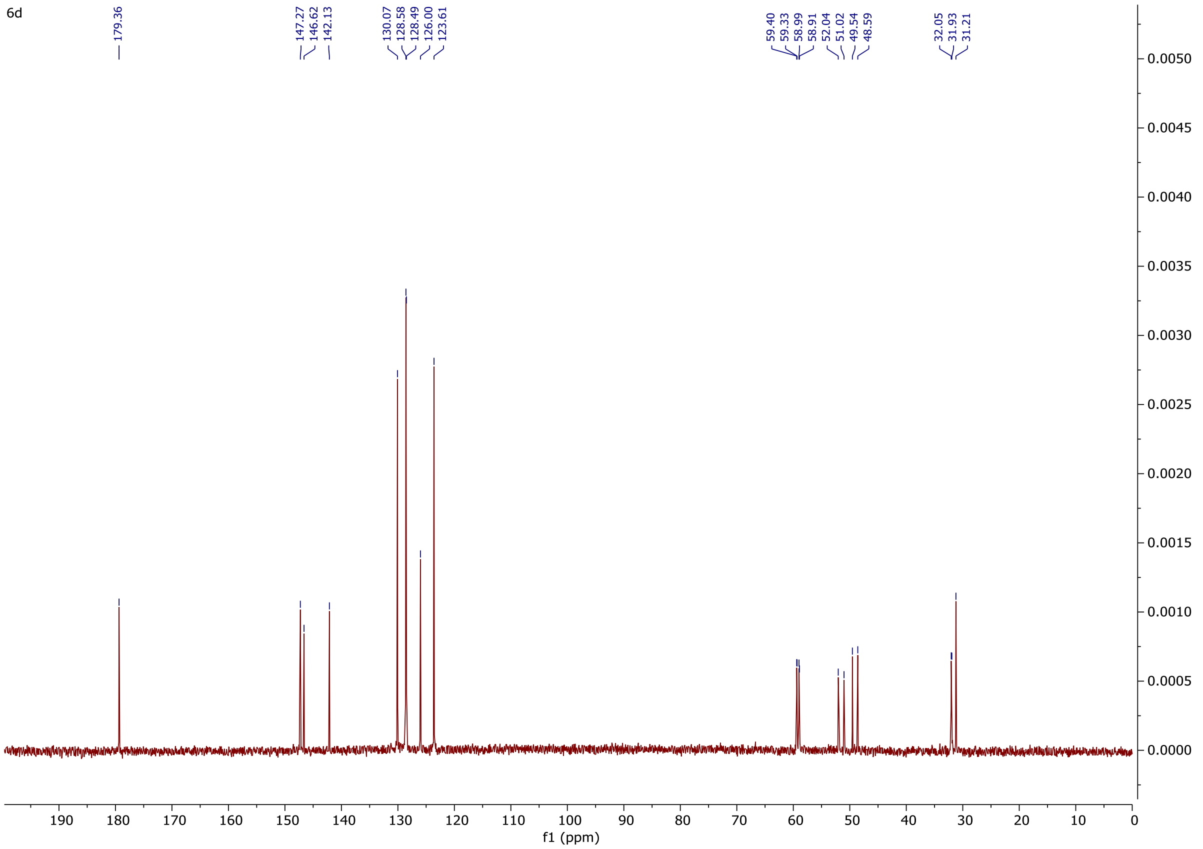

Figure S15. ^{13}C NMR spectrum of compound **6d**

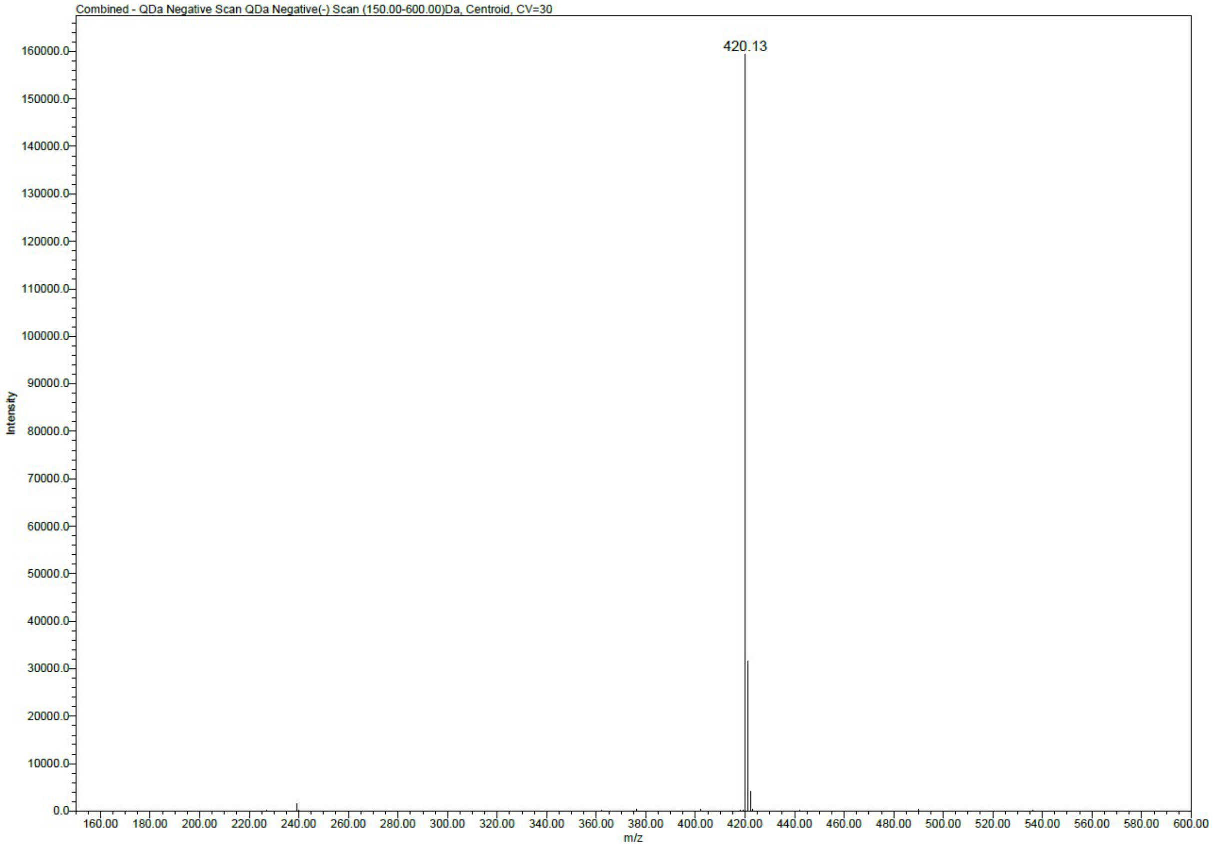

Figure S16. MS spectrum of compound **6d** [M-H]

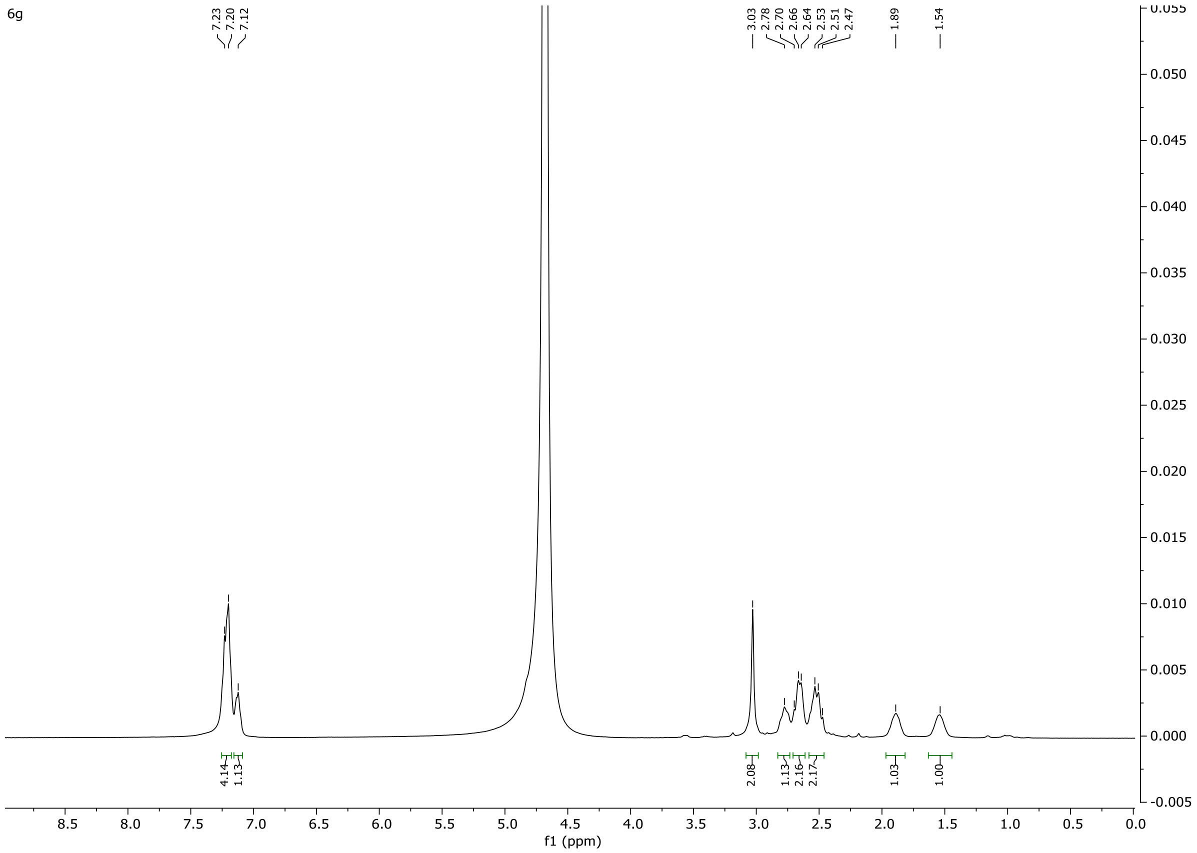

Figure S17. ^{1}H NMR spectrum of compound **6g**

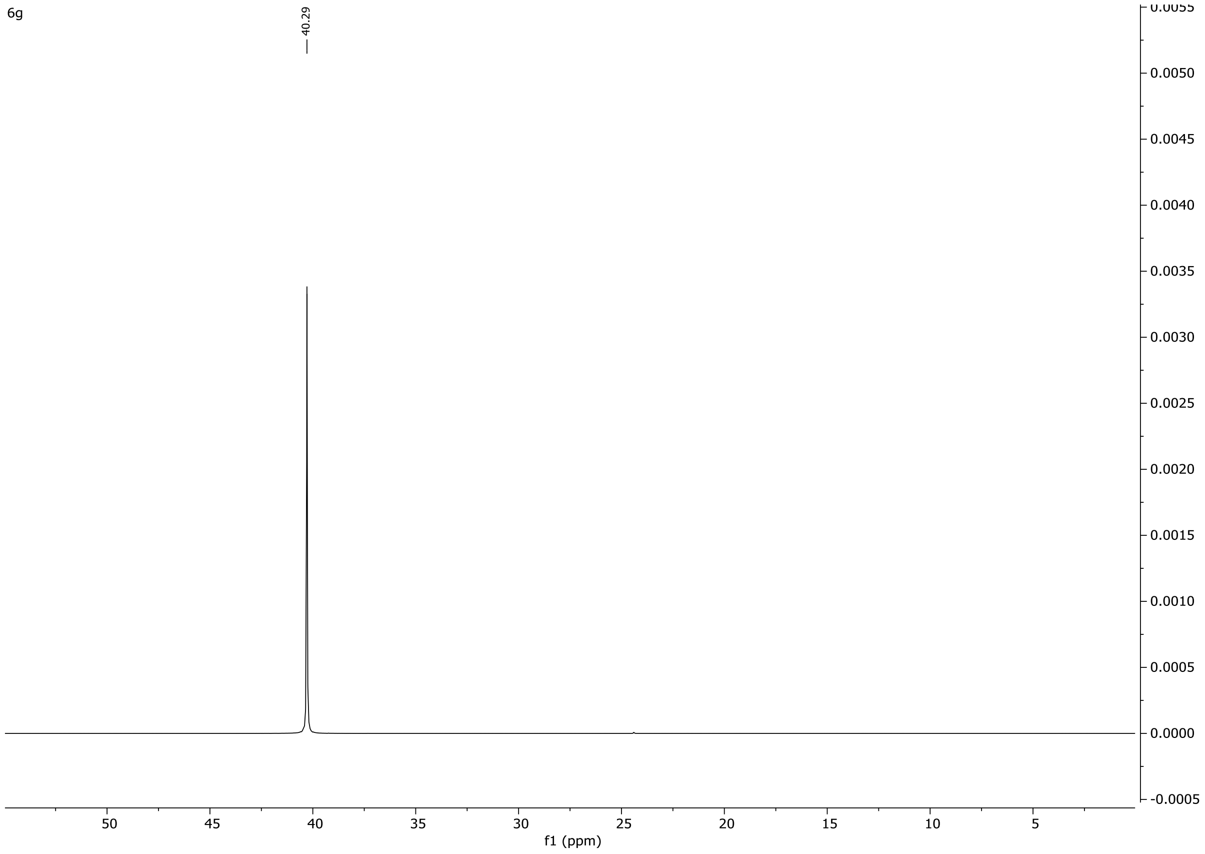

Figure S18. ^{31}P NMR spectrum of compound **6g**

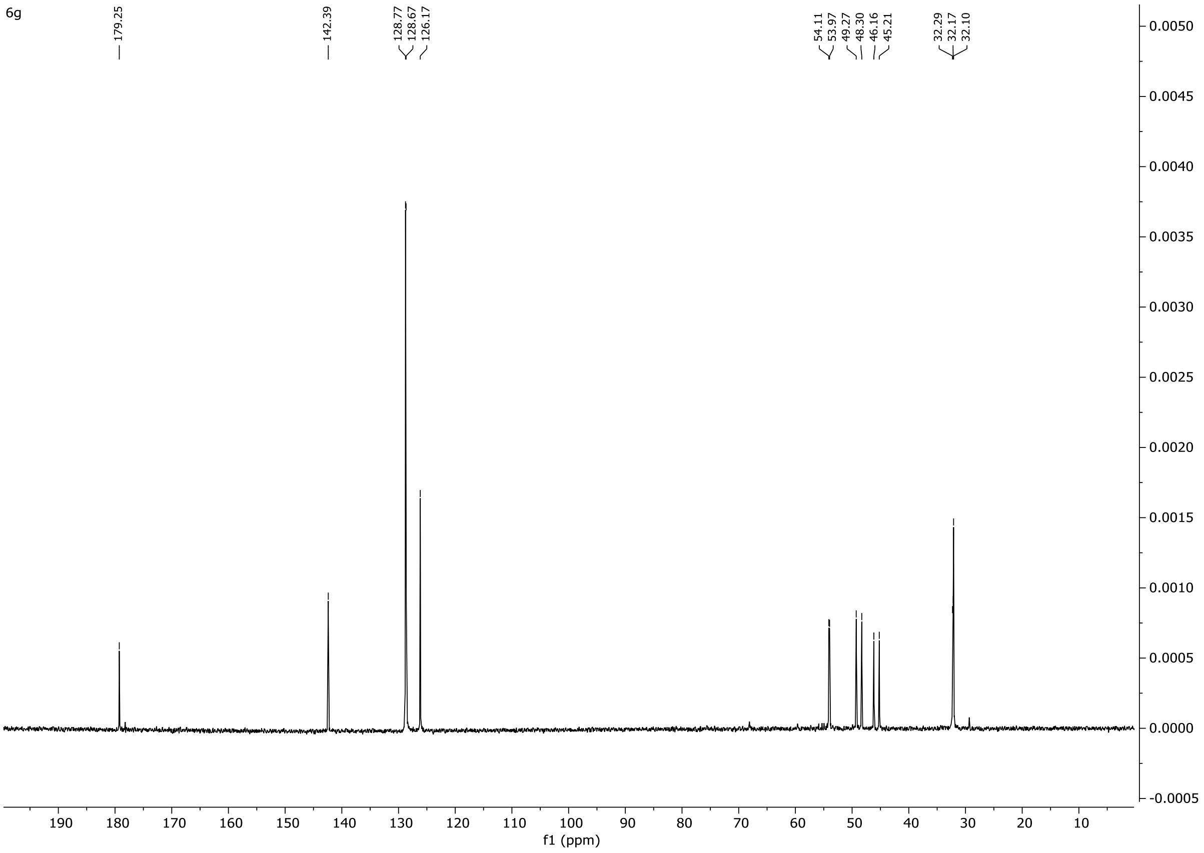

Figure S19. ^{13}C NMR spectrum of compound **6g**

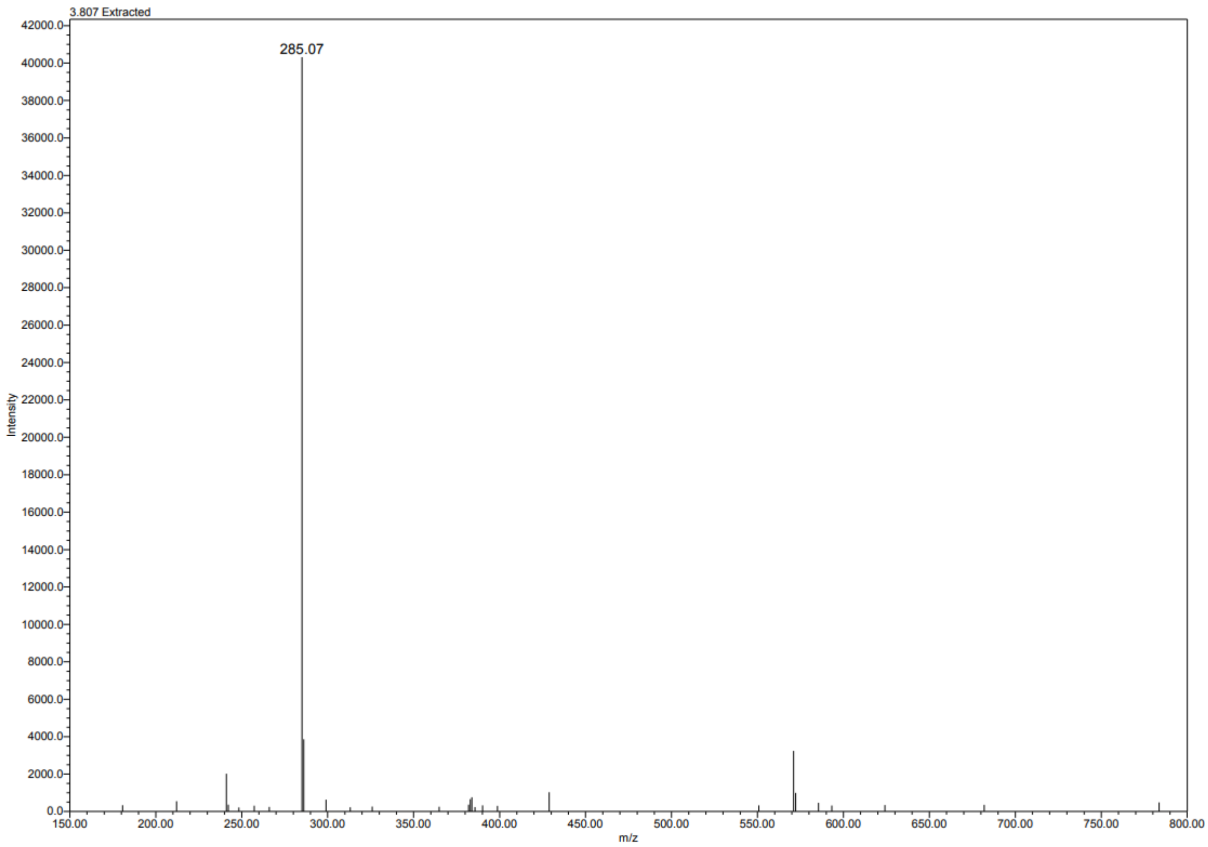

Figure S20. MS spectrum of compound **6g** [M-H]
